# The small heat shock protein B8 (HSPB8) confers resistance to bortezomib by promoting autophagic removal of misfolded proteins in multiple myeloma cells

**DOI:** 10.18632/oncotarget.2193

**Published:** 2014-07-09

**Authors:** Mohamed-Amine Hamouda, Nathalie Belhacene, Alexandre Puissant, Pascal Colosetti, Guillaume Robert, Arnaud Jacquel, Bernard Mari, Patrick Auberger, Frederic Luciano

**Affiliations:** ^1^ INSERM U1065, C3M, Team 2, Nice, France; ^2^ Université de Nice Sophia-Antipolis; ^3^ Equipe labellisée par la Ligue Nationale Contre le Cancer; ^4^ Dana-Farber Cancer Institute and Boston Children's Hospital, Harvard Medical School, Boston, USA; ^5^ UMR7275 CNRS-UNS, Institut de Pharmacologie Moléculaire et Cellulaire, Valbonne, France

**Keywords:** multiple myeloma, velcade, resistance, HSPB8, aggregates, autophagy

## Abstract

Velcade is one of the inescapable drug to treat patient suffering from multiple myeloma (MM) and resistance to this drug represents a major drawback for patients. However, the mechanisms underlying velcade resistance remain incompletely understood. We derived several U266 MM cell clones that resist to velcade. U266-resistant cells were resistant to velcade-induced cell death but exhibited a similar sensitivity to various proapoptotic stimuli. Careful analysis of proteosomal subunits and proteasome enzymatic activities showed that neither the composition nor the activity of the proteasome was affected in velcade-resistant cells. Elimination of velcade-induced poly-ubiquitinated proteins and protein aggregates was drastically stimulated in the resistant cells and correlated with increased cell survival. Inhibition of the lysosomal activity in velcade-resistant cells resulted in an increase of cell aggregates and decrease survival, indicating that aggregates are eliminated through lysosomal degradation. In addition, pangenomic profiling of velcade-sensitive and resistant cells showed that the small heat shock protein HSPB8 was overexpressed in resistant cells. Finally, gain and loss of function experiment demonstrated that HSPB8 is a key factor for velcade resistance. In conclusion, HSPB8 plays an important role for the elimination of aggregates in velcade-resistant cells that contributes to their enhanced survival.

## INTRODUCTION

The ubiquitin-proteasome pathway plays an essential role in the degradation of 80% of ubiquitin-tagged intracellular proteins, many of which play a regulatory role in cell proliferation, cell survival, and signaling processes [1-3]. Degradation of regulatory proteins by this process is essential for maintaining normal cellular function. In cancer cells, proteasome is also required for the tumor cell growth, angiogenesis and apoptosis [4]. Proteasome inhibition has emerged as a new therapeutic option against cancer [5]. Bortezomib (PS341, velcade) is the first proteasome inhibitor that has been approved for the treatment of multiple myeloma (MM), resulting in outstanding response rate in both relapsed/refractory and newly diagnosed MM patients [6, 7]. Velcade is a reversible inhibitor that target primarily the β5-subunit (PSMB5) subunit/chymotrypsin-like proteolytic activity of the 26S proteasome and to a lesser extend the β1 (PSMB6) subunit/caspase-like activity. At higher concentrations, velcade also inhibits the trypsin-like activity harbored by the β2 (PSMB7) proteasome subunit [8]. Velcade disrupts the equilibrium between protein biosynthesis and protein degradation, which results in cell death by apoptosis. Proteasome inhibition leads to the accumulation of damaged proteins in the intracellular environment which causes endoplasmic reticulum overload integrated as an ER stress [9]. Then the cell induces protective responses, so called the unfolded protein response (UPR) that promotes refolding or elimination of damaged proteins. If the accumulation of damaged proteins is excessive, cell can at last triggers apoptosis through CHOP, caspase-4 and caspase-12 activation. It was also reported that proteasome inhibition triggers apoptosis by affecting the levels of many regulatory proteins, resulting in inhibition of nuclear factor κB (NF-κB), increased activity P53 and Bax leading to the activation of c-Jun NH_2_ –terminal kinase (JNK), which in turn activates caspase-8 and caspase-3 [10].

Multiple myeloma (MM) is a plasma cell malignancy [11] that remains incurable despite conventional treatment [12] or high-dose therapy [13]. It has a prevalence of 50 000 patients in the United States, occurring in approximately 16 000 new individuals each year. Autologous stem cell transplantation remains a good option for treating this disease; however, all patients will unavoidably relapse. Therefore, considerable efforts have been devoted to the development of new therapeutic strategies for the treatment of patients suffering from MM. Drugs such as lenalidomide (Revlimid®) and bortezomib (Velcade®) have significantly improved the overall survival rates of patients [14]. Despite promising clinical activity, some patients with MM failed to respond to velcade therapy or respond briefly and relapse [15]. The mechanism of velcade resistance remains puzzling. In lymphoma cells, high constitutive expression of heat shock protein 27 (HSP27) was reported to confer resistance to velcade [16]. In the context of acquired resistance to velcade, two previous studies reported an enhanced cellular efflux via the overexpression of multidrug resistance (MDR) transporter P-glycoprotein (Pgp; ABCB1; MDR1) [17] or multidrug resistance-related protein 1 (MRP1;ABCC1) [18] in ovarian cells. To date, different individual cell lines (including MM cell lines) have been adapted to increased concentrations of velcade [19-24]. In most of these studies, mutations in the active center of the β5 polypeptide have been repeatedly reported (G322A, C323T, C326T), that either lead or not to increased chymotryptic activity with or without overexpression of individual proteasome subunits.

In the present study, we derived several U266 MM cell clones that resist to velcade. U266-resistant cell clones were fully resistant to velcade-induced cell death compared to their sensitive parental counterpart but exhibited a similar sensitivity to a large spectrum of proapoptotic stimuli. Neither the composition nor the activity of the proteasome was affected in velcade-resistant cells. Importantly, elimination of velcade-induced poly-ubiquinated proteins and protein aggregates was drastically stimulated in resistant cells and correlated with an increase in cell survival in the presence of the drug. Inhibition of the lysosomal activity in velcade-resistant cells resulted in a diminution of cell aggregates and increased survival, indicating that protein aggregates are eliminated through lysosomal degradation. In addition, pangenomic profiling of velcade-sensitive and resistant cell lines showed that the small heat shock protein HSPB8 was overexpressed in resistant cells. Finally, gain and loss of function experiment demonstrated that HSPB8 is a key factor for velcade resistance. In conclusion, these findings establish for the first time that HSPB8 plays an important role for the elimination of cell aggregates in velcade-resistant cells that contributes to their enhanced survival.

## RESULTS

### Generation and characterization of multiple myeloma cell lines resistant to velcade

To gain insights into the molecular mechanisms of resistance to conventional therapies in MM, we generated U266 cell clones resistant to velcade, one of the leading drug to treat patients suffering from MM. For that purpose, the U266 cell line was incubated with iterative and increasing concentrations of velcade for a long period of time. After 10 months of selection we generated a bulk of U266 cells that resist up 20 nM velcade. In a first set of experiments, we determined the sensitivity of these cells to velcade using the XTT cell metabolism assay. Supplementary Figure 1A showed the loss of cell metabolism induced by increasing concentrations of velcade on the bulk of U266 cells rendered resistant to 10, 12.5, 15 or 20 nM of this proteasome inhibitor. In the control cells, the IC50 value for velcade was around 2 nM, whereas 10-20 nM of the drug was required to inhibit 50% of cell metabolism in the bulk of U266 cells. After serial dilutions, we obtained several U266 cell clones from the bulk of cells resistant to 20 nM velcade. In the U266 sensitive cell clones, the IC50 value for the velcade-induced loss of cell metabolism was 4-5 nM compared to 25-30 nM in the bulk of U266 resistant cells and clone U266R9 (Supplementary Figure 1B). Two clones namely, U266R6 and U266R11 were found to be highly resistant to velcade, with IC50 values of 80 and 100 nM, respectively.

**Figure 1:**
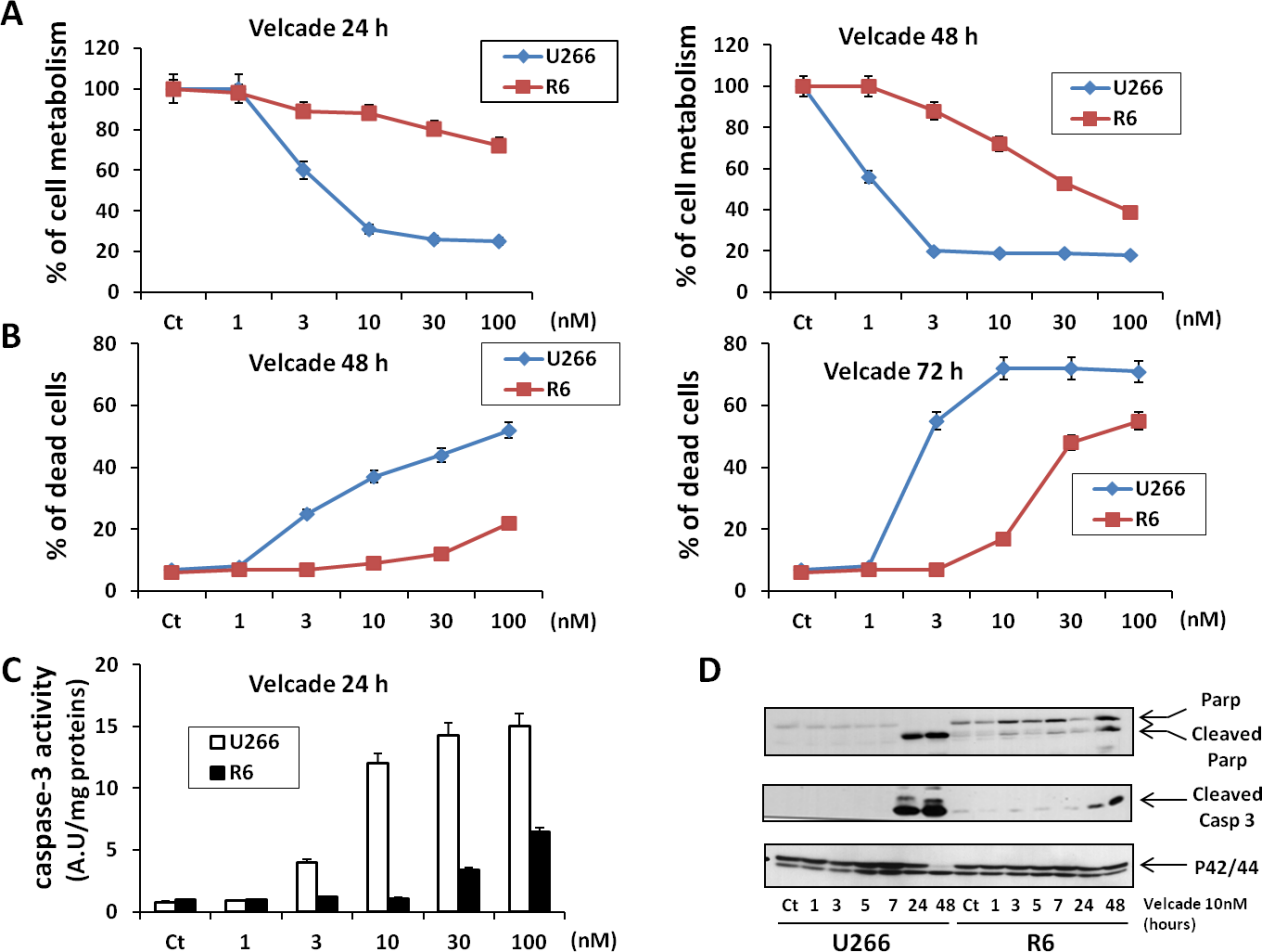
Characterization of a MM cell line resistant to velcade A, U266 and R6 MM cell lines were incubated with increasing concentrations of velcade (1 to 100 nM) for 24 h (left panel) or 48 h (right panel). Then, the cells were collected and cell viability was assessed with the XTT cell metabolism assay. B, U266 and R6 cells were incubated with increasing concentrations of velcade (1 to 100 nM) for 48 h (left panel) or 72 h (right panel). Then, the cells were harvested and incubated with Propidium iodide (PI), and the percentage of dead cells was assessed by flow cytometry. C, U266 and R6 cells were incubated with increasing concentrations of velcade (1 to 100 nM) for 24 h. Then, the cells were collected and lysed in caspase buffer, and caspase activity was assessed using DEVD-AFC as substrate. D, U266 and and R6 cell lines were incubated with velcade (10 nM) for 1 h to 48 h. Then, the cells were collected, washed and lysed, and total protein extracts were subjected to SDS-PAGE immunoblot using anti-Parp, anti-Caspase-3 and anti-P42/44 antibodies.

For all the forthcoming experiments we thus decided to use the U266R6 clone (R6) that exhibited an intermediary resistance (Supplementary Figure 1B). Resistance of the R6 cells to increasing doses of velcade after 24 or 48 h of treatment was further confirmed by the loss of cell metabolism induced by velcade (Figure 1A). Whatever the incubation time, there was a twenty fold increase in the concentration of velcade needed to induce a 50% loss of cell metabolism in R6 cells compared to their U266 counterparts (Figure 1A). There was also an excellent correlation between the loss of cell metabolism in U266 and R6 cells and velcade-mediated cell death assessed by the propidium iodide staining at 48 h (Figure 1B) or the increase in caspase-3 specific enzymatic activity at 24 h (Figure 1C). Accordingly, caspase-3 activation and Poly-ADR Ribose Polymerase (PARP) cleavage were also visualized after 24 h or 48 h of velcade treatment in U266 parental cells, but only poorly detected in their velcade-resistant counterpart (Figure 1D).

### U266 and R6 cells are sensitive to various proapoptotic stimuli but resistant to different proteasome inhibitors

**Figure 2:**
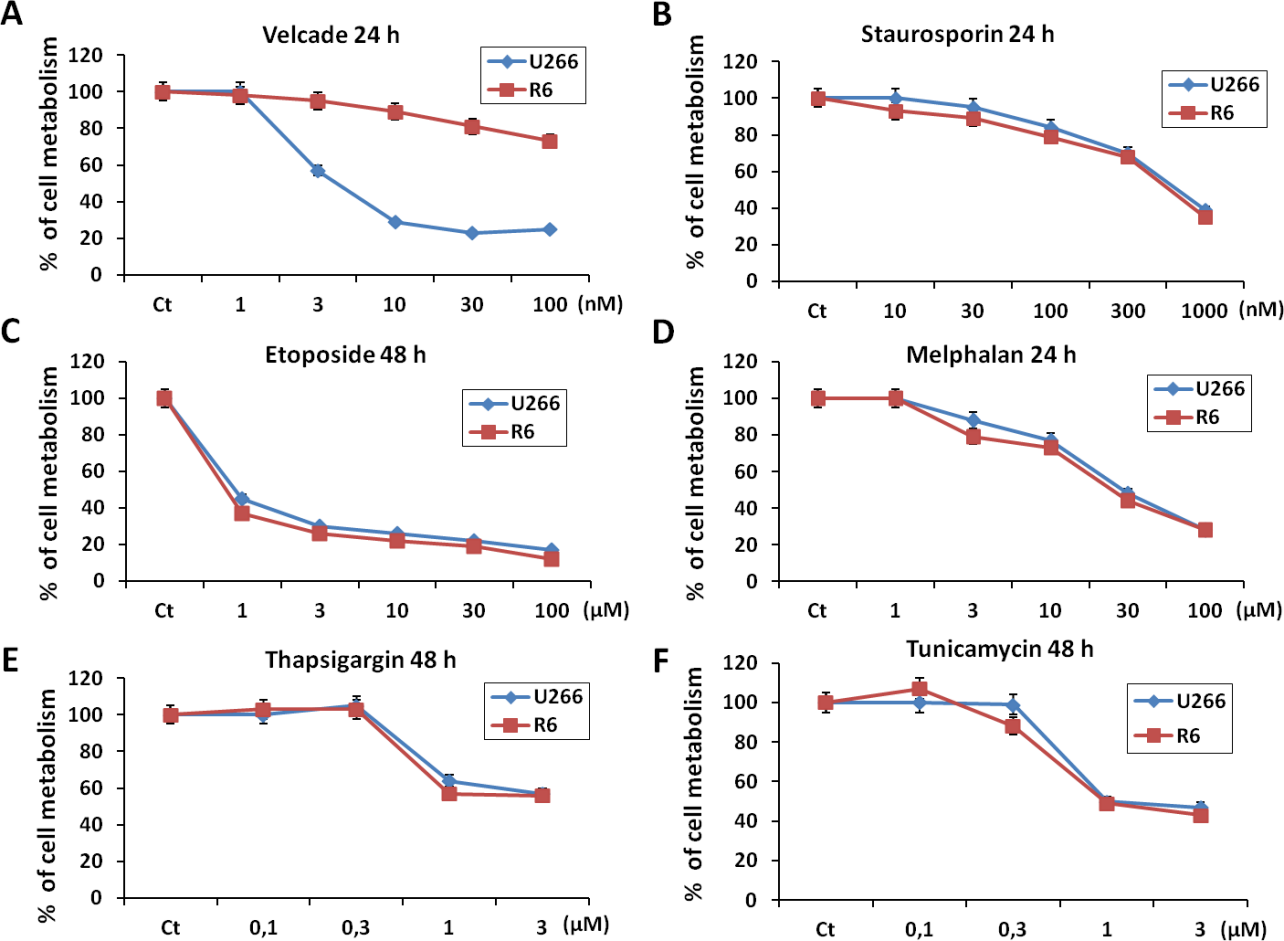
U266 and R6 MM cells are equally sensitive to pro-apoptotic stimuli A to F, U266 and R6 cells were incubated with either velcade (1 to 100 nM), staurosporin (10 nM to 1 μm), etoposide (1 to 100 μM), melphalan (1 to 100 μM), thapsigargin (0.1 to 3 μM) or tunicamycin (0.1 to 3 μM) for the indicated times. Then, the cells were collected, and cell viability was assessed using the XTT cell metabolism assay.

We next investigated the effect of various proapoptotic stimuli on U266 and R6 cells. To this purpose both cell lines were treated with increasing concentrations of either staurosporine or melphalan, an alkylating agent routinely used for the treatment of MM for 24 h. The same cell lines were also incubated with various concentrations of thapsigargin and tunicamycin, two reticulum stress inducing agents or etoposide for 48h, because we established in preliminary experiments that a longer time of treatment with these three later drugs was necessary to induce a significant loss of cell metabolism in velcade-treated cells. As expected, R6 cells exhibited a strong resistance to high concentrations of velcade (Figure 2A). Of note, U266 and R6 cells showed the same sensitivity to all the proapoptotic stimuli used, including melphalan (Figure 2B to F). Therefore, we concluded that R6 cells exhibited a selective resistance to velcade but not to other proapoptotic stimuli.

To investigate whether R6 cells could exhibit a general resistance to proteasome inhibitors, we next look for the effect of clasto-lactacystin, adamanthane (proteosomal β subunit inhibitor), MG132 and epoxomycin in comparison with the effect of velcade. As shown on supplementary Figure 2, besides velcade, R6 cells were also highly resistant to clasto-lacatcystin and MG132 and to a lesser extent to adamanthane and epoxomycin, suggesting a global resistance to a large panel of proteasome inhibitors.

### U266 and R6 cells exhibit the same proteasomal enzymatic activity

**Figure 3:**
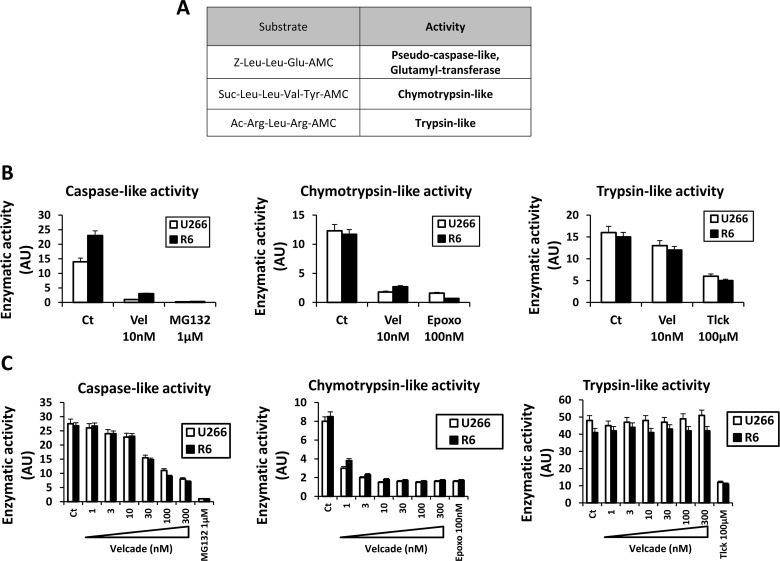
U266 and R6 MM cells exhibit the same global proteasome activity A, Table indicates the sequences of the substrates used to measure the caspase-like and the chymotrypsin-like, and the trypsin-like activities of the proteasome. B, U266 and R6 cells were incubated with either velcade (10 nM), MG132 (1 μM), Epoxomycin (100 nM) or Tlck (100 μM) for 24 h. Then, the cells were collected, washed and lysed, and proteasome activities were measured using the specific AMC conjugated substrates. C, U266 and R6 cells were collected, washed and lysed, and normalized protein extracts were incubated with increasing concentrations of velcade (1 to 300 nM) with either MG132 (1 μM), Epoxomycin (100 nM) or Tlck (100 μM) for 1 h. Then, the cell lysates were collected and the proteasome activities were measured using the specific AMC conjugated substrates.

The proteasome consists on three main enzymatic activities including pseudo-caspase-like / glutamyl transferase, chymotrypsin-like and trypsin-like proteolytic activities that can be assessed using the synthetic fluorescent substrates, Z-Leu-leu-Glu-AMC, Suc-Leu-Leu-Val-Tyr-AMC and Ac-Arg-Leu-Arg-AMC, respectively (Figure 3A). As illustrated on Figure 3B, the global activity (i.e in the absence of velcade) of the proteasome was identical in U266 and R6 cells. The caspase-like and chymotrypsin-like activities were found to be similarly sensitive to velcade and to their specific proteasome inhibitors (MG132 and epoxomycin for the caspase-like and chymotrypsin-like activities, respectively) (Figure 3B left and middle panel). The tryspin-like activity in both cell lines was insensitive to velcade but sensitive to Tlck, an inhibitor of trypsin-like activities (Figure 3B right panel). Finally, we investigated whether small difference in sensitivity to velcade of the different proteolytic activities constitutive of the proteasome could contribute to the resistance of R6 cells to this drug. The dose-response curves for the inhibition by velcade of the caspase-like and chymotrypsin-like activities were superposable in U266 and R6 cells (Figure 3C left and middle panels). In addition, as previously reported, velcade failed to inhibit tryspsin-like activity of the proteasome, (Figure 3C, right panel). In conclusion, our findings clearly demonstrate that the intrinsic activity of the proteasome is identical in U266 and R6 cells. Accordingly, expression of the different protease subunits of the proteasome (PSMB5, PSMB6 and PSMB7) was found to be similar in both cell lines (supplementary Figure 3A). As point mutations in the PSMB5 protein has been previously identified in some velcade-resistant cells [19, 21, 22, 24], we looked for potential mutations in this proteasome subunit. We were unable to detect neither this particular mutation nor any other ones in the PSMB5 subunit present in R6 cells (supplementary Figure 3B). Therefore, we conclude that the nature or the activity of the proteasome is unaffected in our velcade-resistant U266 cell line.

### R6 cells have lower levels of poly-ubiquitinated proteins and aggregates

**Figure 4:**
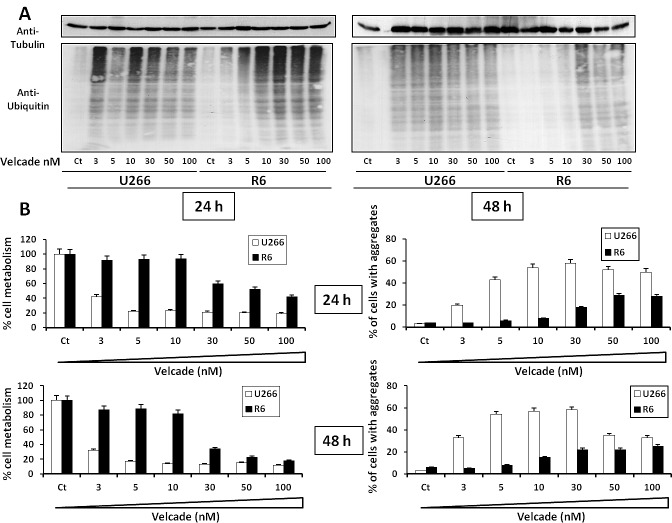
Velcade-treated R6 clone exhibits decreased accumulation of poly-ubiquitinated protein and cellular aggregates A, U266 and R6 cells were stimulated with increasing concentrations of velcade (3 to 100 nM) for 24 h (left panel) or 48 h (right panel). Then the cells were collected and lysed, and normalized protein extracts were subjected to SDS-PAGE and immunoblotting using anti-polyubiquitin and anti-Tubulin antibodies. B, U266 and R6 cells were incubated with increasing concentrations of velcade (3 to 100 nM) for 24 h (upper panels) or 48 h (lower panels). Then, the cells were collected and cell viability was assessed by the XTT cell metabolism assay (left panels). In parallel, the percentage of cells with aggregates was determined by flow cytometry (right panels).

It has been reported previously that Bortezomib (velcade) induced protein aggregate accumulation into the cytoplasm of the RPMI-8226 MM cell line [25]. Thus, the total level of poly-ubiquitaned proteins was evaluated in U266 and R6 cells after 24 or 48 h treatment with increasing concentrations of velcade. For concentrations of velcade ranging from 3 to 10 nM, there was a strong inhibition of poly-ubiquitinated proteins in R6 cells. This was particularly evident in R6 cells treated for 48 h with velcade (Figure 4A). Reduction of poly-ubiquitinated proteins in R6 cells is correlated with a significant gain of cell metabolism after 24 or 48 h in the presence of velcade concentrations ranging from 3 to 30 nM (Figure 4B). Finally, the clearance of intracellular aggregates was increased in R6 cells compared to their sensitive counterpart for the same range of velcade concentrations (Figure 4B). To support the hypothesis that the resistance of R6 cells was due to a higher capacity to eliminate intracellular aggregates, both U266 and R6 cells were stimulated with H_2_0_2_ that is reported to induce cell death and protein aggregates (Supplementary Figure 4). In U266 cells, increasing concentration of H_2_0_2_ induced a loss of cell metabolism (left panel) that was associated with an accumulation of protein aggregates (right panel). In R6 cells, the loss of cell metabolism was dampened and the clearance of protein aggregates was increased. All together our data strongly suggest that an increased clearance of intracellular aggregates allows R6 cells to resist to the effect of velcade.

### Velcade increases the autophagic flux in resistant cells and inhibition of lysosomal activity overcomes resistance to velcade

**Figure 5:**
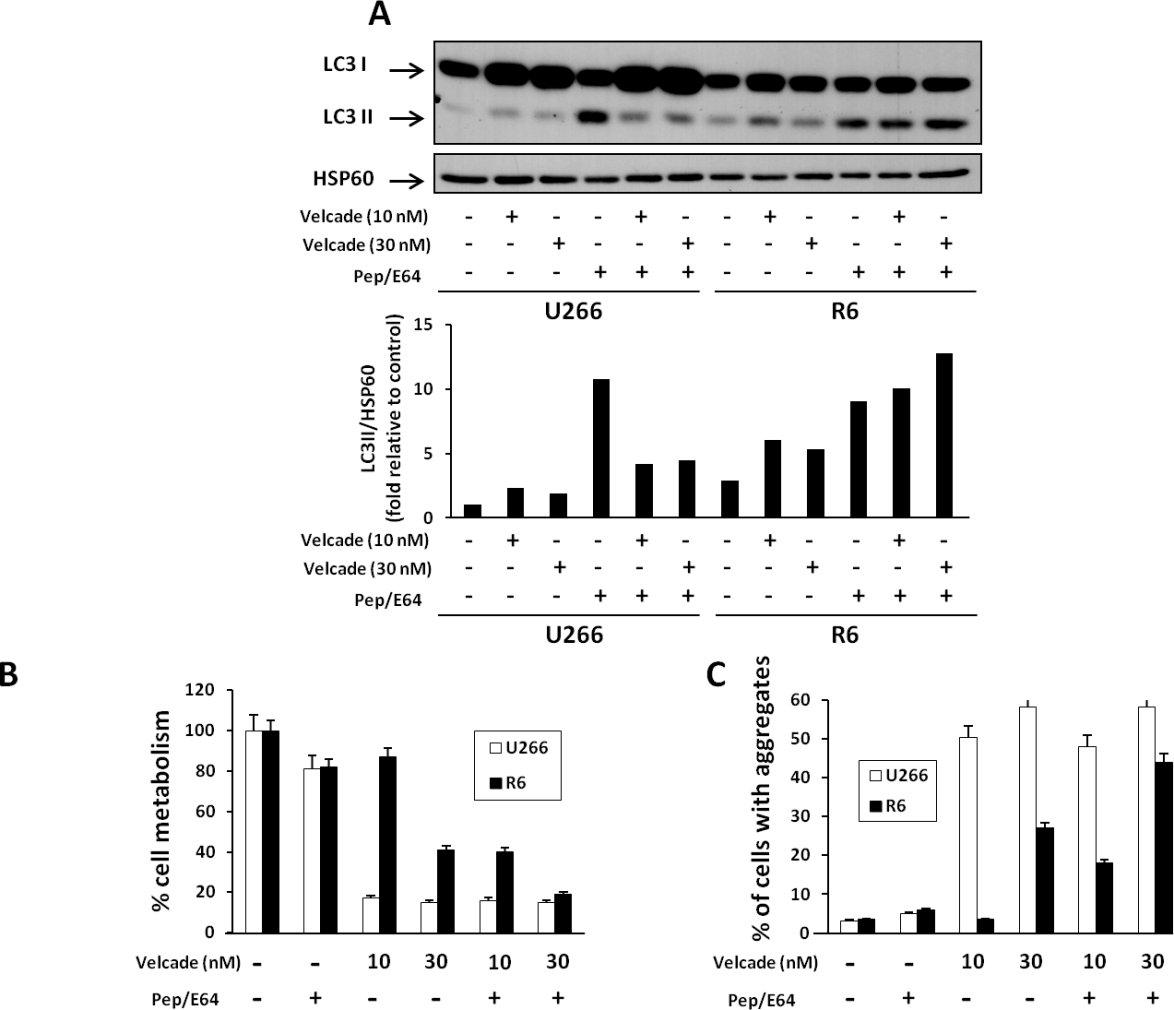
Inhibition of lysosomal degradation restores sensitivity to velcade in R6 cells A, U266 and R6 cells were incubated with increasing concentrations of velcade (10 and 30 nM) in the presence or in absence of the combination of E64 and pepstatin (10 μM) for 24 h. Then, the cells were collected and lysed, and normalized protein extracts were subjected to SDS-PAGE and immunoblotting using anti-LC3 and anti-HSP60 antibodies. B and C, U266 and R6 cells were incubated with increasing concentrations of velcade (10 to 30 nM) in the presence or in absence of the combination of E64 and pepstatin (10 μM) for 24 h. Then, the cells were collected and cell viability was assessed by an XTT cell metabolism assay (B). In parallel, the percentage of cells with aggregates was determined by flow cytometry (C).

Because inhibition of proteasome by velcade triggers the aggresome/autophagy pathway leading to the elimination of protein aggregates by lysosomes, we first analysed the induction of autophagy following velcade stimulation in both U266 clones. The LC3 protein is commonly used as a marker of autophagy. After proteolytic processing by Atg4, LC3 conjugaison with phosphatidylethanolamine allows its insertion into the autophagic vesicule membranes, thus promoting autophagy. The abundance of processed LC3 (LC3-II) serves as a surrogate indicator of autophagic activity. As expected, we observed a slight increase of LC3 processing in both U266 and R6 cells treated with increasing doses of velcade that was nevertheless more robust in the R6 resistant clone (Figure 5A, upper panel). We next investigated whether the R6 cells exhibits an enhanced autophagic flux by using the combination of E64 and Pepstatin that inhibits lysosomal protease activities and consequently induces LC3-II accumulation. We observed an accumulation of LC3-II in both clones treated with E64 and Pepstatin indicating that the basal autophagic flux is similar in U266 and R6 cells. When proteasome and lysosome activities were inhibited by the combination of velcade and E64/Pepstatin respectively, LC3-II accumulation was maintained in the R6 resistant clone, whereas it was inhibited in the U266 parental clone. Quantification of LC3II processing confirmed that when proteasomal and lysosomal activities were inhibited, LC3-II accumulation was dampened in the parental cell line, while it was maintained or ever slightly increased in the resistant clone (Figure 5A). We next analysed whether inhibition of lysosomal activity with the combination of E64 and pepstatin would affect cell viability and clearance of aggregates in U266 and R6 cells treated with increasing doses of velcade. E64 + Pepstatin significantly increased the velcade-induced loss of cell metabolism and accumulation of cell aggregates in R6 cells after 24 h (Figure 5B and 5C). A similar loss of velcade-mediated cell metabolism and increase aggregate accumulation was observed in R6 cells treated with Chloroquine (10 μM), another inhibitor of lysosomal activity (Supplemental 5). R6 cells were also stimulated with increasing concentrations of MG132 in the presence or in absence of either Pep/E64 (Supplemental 6A) or Bafilomycin A1, another inhibitor of lysosomal activity (Supplemental 6B). Percentages of dead cells (upper panels) or cell metabolism assay (lower panels) showed that R6 cells were resistant to MG132 and that inhibition of the lysosomal activity resensitized R6 cells to MG132. Taken together, these data show that a better clearance of cellular aggregates by the aggresome/autophagy pathway upon velcade treatment is responsible for the increase in cell metabolism and the resistance of R6 cells to cell death.

### Pangenomic profiling of U266 and R6 cells

**Figure 6:**
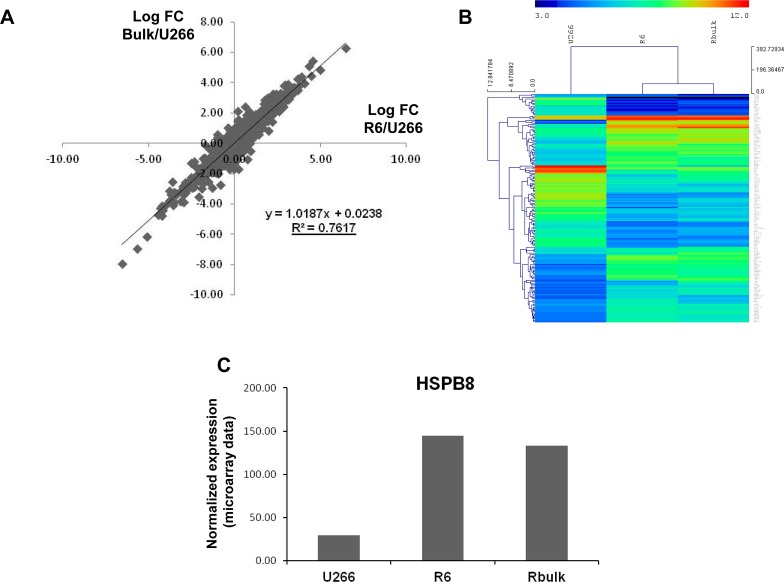
Whole genome profiling of velcade-resistant cells RNA samples were prepared from U266 parental population or velcade-resistant cells (whole bulk and R6 clone) and mRNA profiles were determined with pan genomic arrays. A, Correlation between the Log2 Fold changes calculated between the 2 velcade-resistant cells and the parental population, as indicated. The determination coefficient (R²) was calculated using log2 fold changes from all microarrays probes. B, Heatmap comparing normalized log2 of the ratio between the 2 velcade-resistant cells and the parental population, as indicated. Distance was measured using the Manhattan distance on the matrix of the log2 (ratio) and classification was performed using a complete agglomeration method. C, Normalized expression value of the HSPB8 probe set in the 3 cell populations.

To gain insights into the mechanisms of resistance to velcade we next compared the transcriptome of U266 parental population with both the R6 clone and the initial bulk of resistant cells using whole genome human microarrays. Table S1 illustrated the 160 most down and up-regulated genes in the 2 resistant cell populations in basal conditions. Importantly, as shown on Figure 6A and 6B, we noticed a strong correlation between the distribution of log2 fold change between the 2 resistant cells, strongly suggesting that the gene profile found in the R6 clone is highly representative of velcade resistance. Among the genes modulated in R6 cells we focused on HSPB8, whose expression was increased 5-6 times in R6 cells compared to their parental counterpart in microarray data (Figure 6C). Interestingly, this gene has been shown previously to be important for the clearance of aggregates. This protein functions as a chaperone in association with BAG3, a stimulator of macroautophagy, and favored degradation of unfolded poly-ubiquitinated protein through the lysosomal degradation pathway [26, 27].

### HSPB8 is upregulated in velcade-resistant clones and is a key actor of resistance to this drug

**Figure 7:**
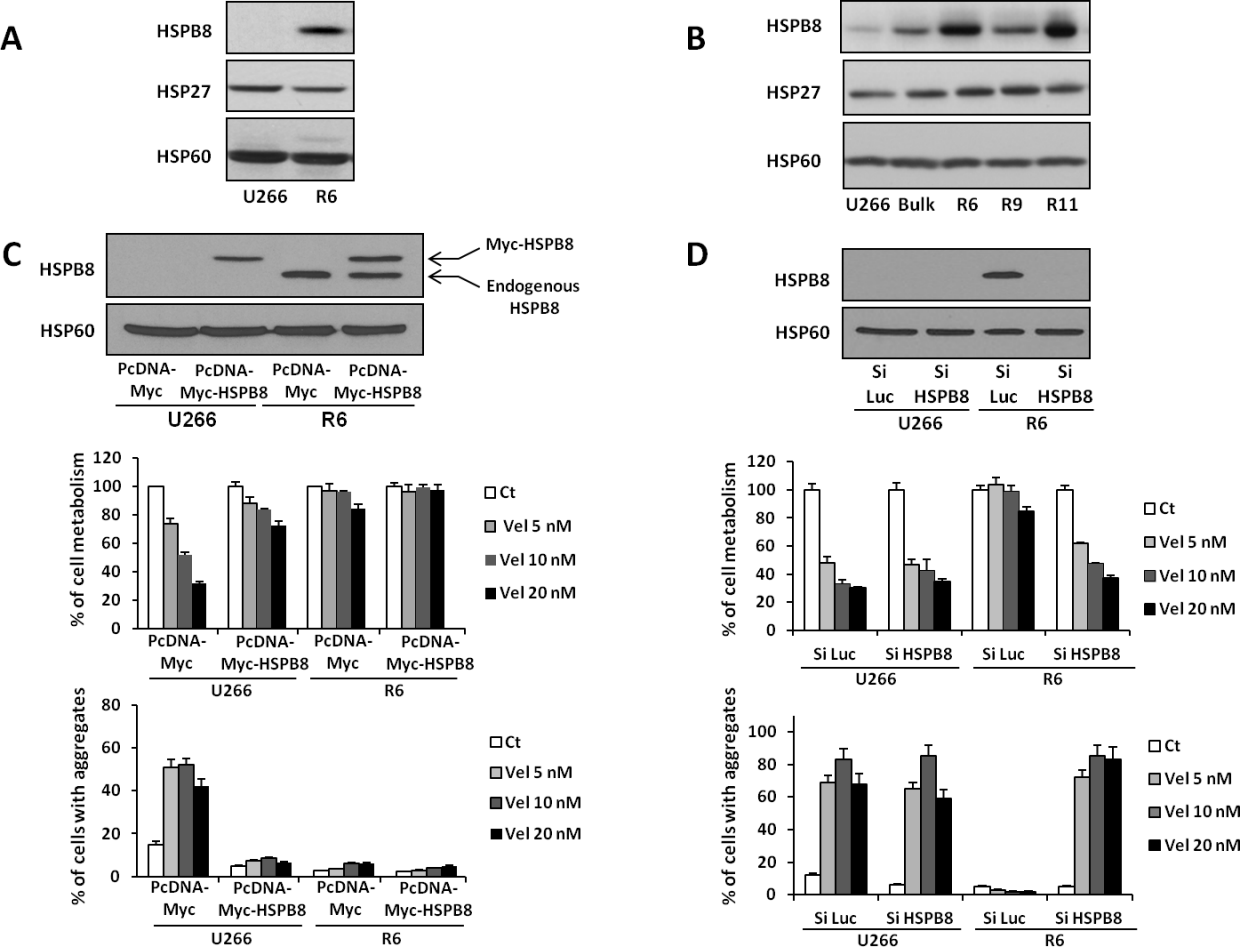
HSPB8 overexpression in R6 cells is responsible for the resistance to velcade A, U266 and R6 cells were collected, washed and lysed, and protein extracts were subjected to SDS-PAGE and immunoblotting using anti-HSPB8, anti-HSP27 and anti-HSP60 antibodies. B, U266 and the various resistant clones obtained previously were subjected to SDS-PAGE and immunoblotting using anti-HSPB8, anti-HSP27 and anti-HSP60 antibodies. C, U266 and R6 cells were transfected with either control PcDNA3-Myc or PcDNA3-Myc-HSPB8 vectors. 48 h later, one fraction of the cells were harvested and lysed. Exogenous Myc-HSPB8 protein (upper band) and endogenous HSPB8 protein (lower band) level was quantified by western blotting using anti-HSPB8 antibody. Correct normalization of protein extracts was confirmed using anti-HSP60 antibody (upper panel). The second fraction of the cells was stimulated with increasing concentrations of velcade (5 to 20 nM) for 24 h. Then, the cells were collected and cell viability was assessed by the XTT cell metabolism assay (middle panel). In parallel, the percentage of cells with aggregates was determined by flow cytometry (lower panel). D, U266 and R6 were transfected with either control siRNA or with HSPB8 siRNA. 48h later, one fraction of the cells was collected and lysed, and HSPB8 protein silencing was confirmed by western blotting using anti-HSPB8 antibody. Normalization of protein extracts was confirmed using anti-HSP60 antibody (upper panel). The second portion of the cells was stimulated with increasing concentrations of velcade (5 to 20 nM) for 24 h. Cell viability was assessed using the XTT cell metabolism assay (middle panel). In parallel, the percentage of cells with aggregates was determined by flow cytometry (lower panel).

Overexpression of the HSPB8 protein was confirmed in R6 cells by western blotting (Figure 7A). Importantly, HSP27 another heat shock protein was present at the same level in U266 and R6 cells (Figure 7A). To disprove that increased expression of HSPB8 could be a clonal particularity of the R6 cell line, we looked for its expression in the bulk of U266 resistant cells and in the different U266 cell clones. As shown on figure 7B, we found a very good correlation between HSPB8 expression in the different clones and their sensitivity to velcade (Figure 7B and Supplementary Figure 1B).

Next, we wanted to determine whether velcade can modulate HSPB8 expression level in U266 and R6 clones. In accordance with the pangenomic profiling, RT-PCR experiment showed an increase in HSPB8 mRNA in R6 cells but not U266 cells in basal conditions (Supplementary Figure 7). In R6 cells, increasing concentrations of velcade led to a strong induction of HSPB8 mRNA, while the proteasome inhibitor induced a slight increase in HSPB8 mRNA expression at low dose of velcade in U266 cells (Supplementary Figure 7A). In R6 cells, induction of HSPB8 upon velcade treatment was also confirmed at the protein level (Supplementary Figure 7B).

This prompted us to investigate further the role of HSPB8 in the resistance to velcade. To this aim, we performed gain and loss of function experiments in U266 and R6 cells, respectively (Figure 7C and D). We first expressed myc-Tagged HSPB8 in U266 cells to a similar level than endogenous HSPB8 in R6 cells (Figure 7C upper panel). Increased expression of HSPB8 in R6 cells further reduced the very weak loss of cell metabolism induced by velcade. Importantly increased expression of HSPB8 in U266 cells significantly protects them from the velcade-induced loss of cell metabolism, whatever the concentrations of the drug used (Figure 7C middle panel). Indeed, transfected U266 cells that expressed the same level of HSPB8 than their R6 counterpart exhibited the same response to velcade. As expected, the resistance of U266 cells to velcade conferred by HSPB8 expression was correlated with a drastic diminution of cell aggregates (Figure 7C lower panel).

To further confirm the implication of HSPB8 in the resistance to velcade, we knocked down its expression in U266 and R6 cells using a siRNA approach. Specific silencing of HSPB8 in R6 cells was first confirmed by Western Blotting (Figure 7D upper panel). HSPB8 silencing failed to affect the velcade-mediated loss of cell metabolism in U266 cells conversely to cells treated with the control siLuc, indicating the lack of global toxicity of the HSPB8 siRNA. Importantly, HSPB8 knock down in R6 cells restored sensitivity to velcade whatever the concentrations used. At the higher concentrations of velcade, the loss of cell metabolism reached two third of the velcade effect in U266 cells (Figure 7D middle panel). Notably, resensitization of R6 cells to velcade was closely linked to a huge increase in cell aggregates (Figure 7D lower panel). Gain and loss of function experiments were also confirmed in the R11 resistant clone (Supplementary Figure 8).

To confirm that HSPB8 overexpression inhibits specifically the proteasome inhibitor-induced loss of cell metabolism, we first expressed myc-Tagged HSPB8 in U266 and R6 cells and stimulated clones with either increasing concentrations of different proteasome inhibitors or with various proapoptotic stimuli (Supplemental Figure 9). As expected, in basal conditions, while R6 cells exhibited a strong resistance to the different proteasome inhibitors (panels A, B and C), U266 and R6 cells showed the same sensitivity to all the proapoptotic stimuli used (panels D, E and F). HSPB8 overexpression in U266 cells dampened the proteasome inhibitor-induced loss of metabolism (panels A, B and C) while it had no effect on proapoptotic stimuli-induced loss of cell metabolism (panels D, E and F). Importantly, in R6 cells, HSPB8 overexpression reduced slightly the loss of cell metabolism induced by proteasome inhibitors but had no effect on proapoptotic stimuli-induced cell metabolism. Overexpression of the myc-Tagged HSPB8 protein was confirmed in U266 and R6 cells by western blotting (panel G). Therefore, we concluded that overexpression of HSPB8 conferred a selective resistance to proteasome inhibitors but not to other proapoptotic stimuli.

Taken together our results highlight the key role of HSPB8 in the resistance to velcade that is in agreement with its capability to clear intracellular aggregates.

## DISCUSSION

The newly established velcade-resistant U266 MM cell lines described in the present report exhibited a strong cross-sensitivity to a wide panel of proteasome inhibitors but remained sensitive to various proapoptotic stimuli. These results indicate that R6 clones exhibit a selective resistance to proteasome inhibitors. In the context of acquired resistance to velcade, previous studies reported an enhanced cellular efflux via the overexpression of multidrug resistance (MDR) transporter P-glycoprotein (Pgp) [17] or multidrug resistance-related protein 1 (MRP1) [18]. We excluded this possibility because i) pangenomic profiling of velcade-sensitive and resistant cell lines didn't highlight an overexpression of PgP transporter or MRP1 protein ii) parental U266 cells and the different resistant clones described herein were similarly sensitive to cell death inducers that act independently of the proteasome inhibition. Resistance to etoposide or thapsigargin was also reported to be linked to Pgp and MRP1 overexpression respectively [28, 29]. This is not the case in our velcade resistant cell lines in which are sensitive to both effectors.

To investigate the mechanisms of velcade resistance that occur frequently in hematopoietic malignancies, several studies were conducted in velcade adapted MM, myelomonocytic, promyelocytic, T lymphoblastic lymphoma/leukemia or Burkitt lymphoma cells cell lines [19-24, 30]. Authors reported mutations in the active center of the β5 polypeptide (PSMB5), which either lead or not to increased chymotryptic activity with or without overexpression of individual proteasome subunits. However, to the best of our knowledge, no evidence of mutations or overexpression of the PSMB5 proteasome subunit has been reported in any case of myeloma patient [31-34]. These results show that in myeloma patients resistant to velcade, the direct implication of a proteasome defect remains to be established.

In our models velcade resistant U266 MM clones we failed to observe mutations or overexpression of the PSMB5 core leading to an increased chymotryptic activity. Moreover, no mutations or overexpression of the PSMB6 or PSMB7 proteasome subunits that could explain a reinforced caspase-like and trypsin-like activities, were detected (Supplemental 3 and data not shown). Using synthetic fluorescent substrates, we also measured the caspase-like, chymotrypsin-like and trypsin-like proteolytic activities in either U266 or R6 cells or in the corresponding cell lysates incubated or not with velcade. We demonstrated that both U266 and R6 clones exhibited similar basal chymotrypsin-like and caspase-like proteasome activities that were both sensitive to inhibition by velcade. In conclusion, our findings rule out the possibility that a modification in the intrinsic activity of the proteasome can explain the resistance of the R6 clones to velcade.

One mechanism that could explain the difference between our results and the one obtained from other groups could be the concentrations of velcade used to generate these U266 resistant clones. In the previous studies, the authors used concentrations of velcade up to 1 μM to select their clones [19, 21, 22, 24]. In our case, we did not exceed 20 nM because this concentration corresponds to the dose commonly administered to patients (1.3 mg/m^2^) [35]. It is thus reasonable to assume that different resistance mechanisms are at play in the cells treated with low and high doses of velcade.

The aggresome/autophagy pathway is an important process that is critical for the degradation of a plethora of proteins via the lysosomes. This process is further involved when the proteasome/ubiquitin pathway is inhibited [36]. In our model, we observed accumulation of both poly-ubiquitinated and protein aggregates in both U266 and R6 cell lines stimulated with increased concentrations of velcade (Figure 4). These results confirmed that both cell lines engaged a similar upstream molecular response following velcade challenging. Then, we showed that elimination of velcade-induced poly-ubiquitinated proteins and protein aggregates was drastically stimulated in resistant cells and correlated with an increased cell survival after 48 h in the presence of the drug. Inhibition of the lysosomal activity with the combination of E64 and Pepstatin (Figure 5B and C) or Chloroquine (Supplemental 5) in velcade-resistant cells resulted in an increase of cell aggregates and a decreased survival, indicating that protein aggregates are eliminated through the lysosomal pathway. Moreover, we showed that resistant cells engaged a more robust induction of autophagic flux upon velcade treatment compared to their parental counterpart (Figure 5A and B). These results are in agreement with findings from the literature reporting that i/ Proteasome inhibition by velcade resulted in the accumulation of ubiquitinated protein leading to protein aggregation and proteotoxicity [25] ii/ Inhibition of the proteasome by velcade triggers the aggresome/autophagy pathway leading to the elimination of protein aggregates by lysosomes [36, 37] and iii/ Inhibition of proteasome and aggresome functions induces a synergistic antitumor activity in MM [10, 38, 39]. To our knowledge and importantly, this is the first description of a MM model of velcade adapted-cells that reveals an enhance capacity to degrade toxic protein aggregates through the aggresome/autophagy pathway.

To investigate further the mechanism of resistance to velcade, we next analyzed the alteration of gene expression in U266 and R6 cells using an Affymetrix pangenomic profiling. Heat Shock Proteins (HSP) have been extensively described to be implicated in protein quality control and the removal of misfolded proteins [40]. The main machinery involves molecular chaperones such as HSP70, HSP90 and small heat shock proteins (also called HSPB) which can recognize and bind unfolded proteins thereby preventing their aggregation. The fate of their bound substrates (renaturation or degradation by the aggresome/autophagy pathway) depends on the interaction of HSP70 with specific cochaperones such as the E3 ligase CHIP and the BAG domain containing proteins [41, 42]. We thus more specially focused on HSP protein family expression in velcade-sensitive and resistant clones. From the pangenomic study, we determined that among the HSP protein members, only the transcript encoding the Small Heath Shock Protein B8 (HSPB8, HSP22, H11, E2IG1) was overexpressed in R6 cells compared to their parental counterpart. Of note, gain and loss of function experiments demonstrated that HSPB8 is a key factor for velcade resistance in our cell line model. In association with BAG3 and HSP70, the chaperone activity of HSPB8 was reported to be involved in the delivery of misfolded proteins to the macroautophagy machinery [26, 27]. Thus, the function of HSPB8 in velcade-resistant cells is in agreement with the increased autophagy flux and the exacerbated capacity to remove aggregates observed in R6 cells. Induction of BAG3, HSP70 and HSPB8 at both the RNA and protein levels was reported under stress conditions including proteasome inhibition [43, 44]. In our model, we observed an induction of BAG3 and HSP70 protein levels in both U266 parental and R6 cells upon velcade treatment. HSPB8 protein induction was only observed in R6 clone treated with velcade (data not shown) reinforcing the importance of HSPB8 in the process of resistance to velcade.

HSPB8 gene alteration and mutations have been reported in neurological disease. In this context, inactivating HSPB8 mutations (K141E, K141N) were described to cause distal motor neuropathy [45]. In contrast, overexpresion of HSPB8 was reported to prevent the aggregation of Htt43Q, a pathogenic form of Huntingtin responsible for Hungtington's disease [46]. HSPB8 was also shown to promote autophagic removal of misfolded proteins involved in amyotrophic lateral sclerosis (ALS) [47]. Accumulation of aggresomes represents a novel mechanism by which to induce apoptosis, especially among cells where protein production and catabolism are critically important for malignant cell survival, as it is the case for malignant and normal immunoglobulin-producing plasma cells [37, 48]. Thus, it is likely that in MM, HSPB8 up-regulation occurs as a plausible strategy for malignant plasmocytes to counteract the cytotoxic effect of velcade. The HSPB8 protein level in patients suffering MM responding or relapsing to velcade treatment remains to be measured to verify this hypothesis.

In conclusion, the results presented herein suggest that overexpression of HSPB8 in myeloma cells could play an important role for the elimination of cell aggregates in velcade-resistant cells that therefore contributes to their enhanced survival.

## Materials and Methods

### Cell lines

The U266 human MM cell line was purchased from the ATCC and were grown at 37°C under 5% CO2 RPMI medium (Gibco BRL, Paisley, UK) supplemented with 10% fetal calf serum (Gibco BRL, 10270), 50 units/ml penicillin, 50 mg/ml streptomycin (Lonza, DE17-602E) and 1 mM sodium pyruvate (Lonza, BE13-115E).

### Establishment of velcade resistant cell line

From the parental U266 cell line, we established resistant clones by addition in the culture medium of increasing concentrations of velcade (up to 20 nM) for 10 months. From the bulk corresponding to the final step of selection with velcade (20 nM), we isolated 12 clones by limiting dilution. For convenience, we focalized our study onto 3 representative clones (U266R6, U266R9 and U266R11).

### Reagents and antibodies

Sodium fluoride (S7920), sodium orthovanadate (220590), phenylmethylsulfonyl fluoride (PMSF) (P7626), aprotinin (A162-B), leupeptin (SP-04-2217-B), Triton X-100 (N150), Earle's Balanced Salt Solution (EBSS) (E3024), thapsigargin (T9033), tunicamycin (T7765), E64 (E3132), melphalan (M2011), and staurosporin (S4400) were purchased from Sigma. MG132 (BML-PI102), Epoxomycin (BML-PI127) or Tlck (BML-PI121), Z-Leu-Leu-Glu-AMC (BML-ZW9345), Suc-Leu-Leu-Val-Tyr-AMC (BML-P802) and Ac-Arg-Leu-Arg-AMC (BML-AW9785) were from Enzo Life Sciences. Pepstatin (EI-9-B) was obtained from Euromedex. Anti-Hsp60 (sc-1722), anti-Ubiquitin (sc-8017), anti-Hsp27 (sc-1048) and anti-Hsp90 (sc-69703) were purchased from Santa Cruz Biotechnology. Anti-PSMB5 (PW8140), anti-PSMB6 (PW8145) and anti-PSMB7 (PW8895) were from Enzo life sciences. Anti-PARP (9542), anti-HspB8 (30595), anti-LC3 (2775) and Hrp conjugated anti-rabbit (7074) antibodies were obtained from Cell Signaling Technology. Hrp-conjugated anti-mouse (P0260) and anti-goat (P0449) antibodies were from Dakopatts. Cleaved caspase 3 (9661) was purchased from Ozyme.

### Immunoblotting

After stimulation, the cells were lysed at 4°C in lysis buffer (50 mM HEPES pH 7.4, 150 mM NaCl, 20 mM EDTA, 100 μM NaF, 10 μM Na_3_VO_4_, 1 mM PMSF, 10 μg/ml leupeptin, 10 μg/ml aprotinin, and 1% Triton X-100). The lysates were centrifuged at 16,000 ×*g* for 15 min at 4°C, and the supernatants were supplemented with concentrated SDS sample buffer. A total of 30 μg of protein was separated on a 12% polyacrylamide gel and transferred onto a PVDF membrane (Immobilon-P, Millipore, IPVH00010) in a 20 mM Tris, 150 mM glycine and 20% ethanol buffer at 250 mA for 1 h 30 min at 4°C. After blocking the non-specific binding sites in saturation buffer (50 mM Tris pH 7.5, 50 mM NaCl, 0.15% Tween, and 5% BSA), the membranes were incubated with the specific antibodies, washed three times using TNA-1% NP-40 (50 mM Tris pH 7.5, and 150 mM NaCl) and incubated further with HRP-conjugated antibody for 1 h at room temperature. The immunoblots were revealed using the enhanced chemiluminescence detection kit (Pierce, 32106).

### Knock down by siRNA

Stealth small interfering RNAs (siRNA) targeting HSPB8 (HSS178150), were purchased from Invitrogen. Transfection of U266 cells was performed as described previously [49] using the Nucleofector system (Lonza, VCA-1003). Briefly, 2.5 millions of cells were electroporated with either control or HSPB8 siRNA (100 nM) using nucleofector (kit C and program X-05). Then, the cells were plated in 5 ml of RPMI 10% FCS media and incubated for 48 h at 37°C until experiment analysis.

### HSPB8 transfection

PcDNA-Myc-HSPB8 plasmid was kindly provided by Dr Jacques Landry (Centre of recherche cancerologie, University of Laval, Canada). Briefly, 3 millions of U266 and R6 cells were electroporated with 2 μg of either PcDNA-Myc or PcDNA-Myc-HSPB8 vectors using nucleofector (kit C Lonza, VCA-1003 and program X-05). Then, the cells were plated in 5 ml of RPMI 10% FCS media and incubated for 48 h at 37°C until experiment analysis.

### RNA preparation

Total RNA were prepared from the U266 parental cell line, the R6 clone and the initial bulk of resistant cells using TRIzol reagent according to the manufacturer's instructions (Invitrogen). Total RNA (1 μg) was reverse transcribed into cDNA using Superscript II reverse transcriptase (Invitrogen).

### Microarrays experiment

Microarray analyses were performed on the GeneChip Human Gene 1.0 ST Array (Affymetrix, Santa Clara, CA 95051, USA), according to the manufacturer's instructions. RNA from each of the 3 cell population were labeled and hybridized. The experimental data will be deposited in the NCBI Gene Expression Omnibus (GEO) (http://www.ncbi.nlm.nih.gov/geo/). Normalization of microarray data was performed using the Limma package available from Bioconductor (http://www.bioconductor.org). using the RMA method and means of ratios from velcade-resistant cells *versus* U266 parental cells were calculated.

### Measurement of cell metabolism (XTT)

U266 cells or R6 clones were incubated in a 96-well plate with the indicated concentrations of cell death inducers for 24 or 48 h. 50 μl of the XTT reagent (Roche Applied Science, 11-465-015) (sodium 3'-[1-(phenylaminocarbonyl)-3,4-tetrazolium]-bis(4-methoxy-6-nitro) benzene sulfonic acid hydrate) was added to each well. The assay is based on the cleavage of the yellow tetrazolium salt XTT to form an orange formazan dye by metabolically active cells. The absorbance of the formazan product, reflecting cell viability, was measured at 490 nm. Each assay was performed in triplicate.

### Cell Death assay

Cell viability was measured using the propidium iodide (PI) dyed exclusion assay. Briefly, after treatment, the cells were collected and incubated with PI (10 μg/ml) for 5 min. The percentage of PI positive cells was next analysed by flow cytometry using MACSQUANT Analyser (Myltenyi Biotech, 130-092).

### Protein aggregates

Measurement of protein aggregates was performed using the ProteoStat Aggresome Dectection Kit (ENZ-51035-K100) from ENZO Life Sciences according to the manufacturer procedure. Briefly, after stimulation, cells were collected, washed with PBS and centrifugated (400 ×g for 5 min), and fixed with formaldehyde (4%) for 30 min at RT. Then, the cells were centrifugated (800 ×g for 10 min), washed in PBS, and permeabilized (0.5% Triton X-100, 3 mM EDTA, pH 8) for 30 min on ice. The cells were centrifugated again (800xg for 10 min), washed in PBS and incubated with the ProteoStat Aggresome Red Detection Reagent for 30 min at RT. Protein aggregates were measured by flow cytometry in the FL3 channel.

### Measurement of Proteasome activity

U266 and R6 cells were stimulated with velcade for 24 h in the presence or the absence of MG132 (1 μM), Epoxomycin (100 nM) or Tlck (100 μM). Then, the cells were collected, washed, and lysed for 30 min at 4°C in a ATP-containing lysis buffer (50 mM HEPES pH 7.8, 5 mM ATP, 0.5 mM DTT, 5 mM MgCl_2_ and 0.2% Triton X-100). Cell lysates were cleared at 16,000 ×g for 15 min at 4°C. Normalized protein lysates (10 μg/well) were incubated in a 96-well plate with 0.1 mM of either Z-Leu-Leu-Glu-AMC, Suc-Leu-Leu-Val-Tyr-AMC or Ac-Arg-Leu-Arg-AMC to measure caspase-like, chymotrypsin-like and trypsin-like activities, respectively. Proteasome activities were measured by following emission at 460 nm (excitation at 390 nm). Each experiment was performed in quadruplicates and repeated at least four times.

### Measurement of Proteasome activity in cell lysates

U266 and R6 cells were collected and lysed, and normalized protein extracts (10 μg/well) were incubated in a 96-well plate with either velcade (10 nM), MG132 (1 μM), Epoxomycin (100 nM) or Tlck (100 μM) for 3 h. Then, proteasome activities were measured as described above.

### Caspase activity

Following treatments, cells were lysed for 30 min at 4°C in lysis buffer (50 mM HEPES pH 7.4, 150 mM NaCl, 20 mM EDTA, 1 mM PMSF, 10 μg/ml leupeptin, 10 μg/ml aprotinin and 0.2% Triton X-100), and lysates were cleared at 16,000 ×g for 15 min at 4°C. Each assay (in triplicate) was performed with 50 μg of protein prepared from control or stimulated cells. Briefly, cellular extracts were then incubated in a 96-well plate, with 0.2 mM of Ac-DEVD-AMC as substrates for various times at 37°C as previously described. Caspase activity was measured by following emission at 460 nm (excitation at 390 nm) in the presence or in absence of 10 μM of Ac-DEVD-CHO. Each experiment was performed in triplicates and repeated at least four times.

## SUPPLEMENTARY MATERIALS AND METHODS FIGURES AND TABLE


