# Supplementary material for: The small heat shock protein B8 (HSPB8) confers resistance to bortezomib by promoting autophagic removal of misfolded proteins in multiple myeloma cells
Source: Oncotarget. 2014 Jul 9;5(15):6252–66. doi: 10.18632/oncotarget.2193 (PMC4171627; doi:10.18632/oncotarget.2193)
Supplement: Supplementary file 1 [file oncotarget-05-6252-s001.pdf]

The small heat shock protein B8 (HSPB8) confers resistance to bortezomib by promoting autophagic removal of misfolded proteins in multiple myeloma cells

Supplementary Materials and Methods

Amplification of HSPB8 transcript

Total RNA was prepared from the velcade treated U266 and R6 cell lines using TRIzol reagent according to the manufacturer's instructions (Invitrogen). Total RNA (1 µg) was reverse transcribed into cDNA using Superscript II reverse transcriptase (Invitrogen). 2 µl of each cDNA products of velcade treated U266 and R6 cells were amplified using 2 units of Go Taq DNA Polymerase (Promega) in the buffer provided by the manufacturer (1x Green GoTaq Flexi Buffer, 1,5 mM MgSO4, 1 mM dGTP, 1 mM dTTP, 1 mM dCTP, 1 mM dATP) and the HSPB8 primers (sens 5'-GAATTCATGGCTGACGGTCAGATGC-3') and (antisens 5'-CTCGAGTCAGCAGACCCTCTGGGG-3'). Reactions were carried out in the T3000 Thermocycler (Biometra). The mixes were incubated at 95°C for 3 min for the denaturation. Then, the PCR cycling parameters used with the Go Taq DNA polymerase are as follows: denaturation at 95°C for 30 sec, annealing at 65°C for 30 sec and extending at 72°C for 30 sec for 27 cycles. These cycles was followed by a final extension at 72°C for 10min. Actin was used as loading control (25 cycles) using Actin primers (sens 5'- CATGTACGTTGCTATCCAGGC-3') and (antisens 5'-CTCCTTAATGTCACGCACGAT-3'). Finally, each PCR product was analyzed by electrophoresis in 2% agarose gel.

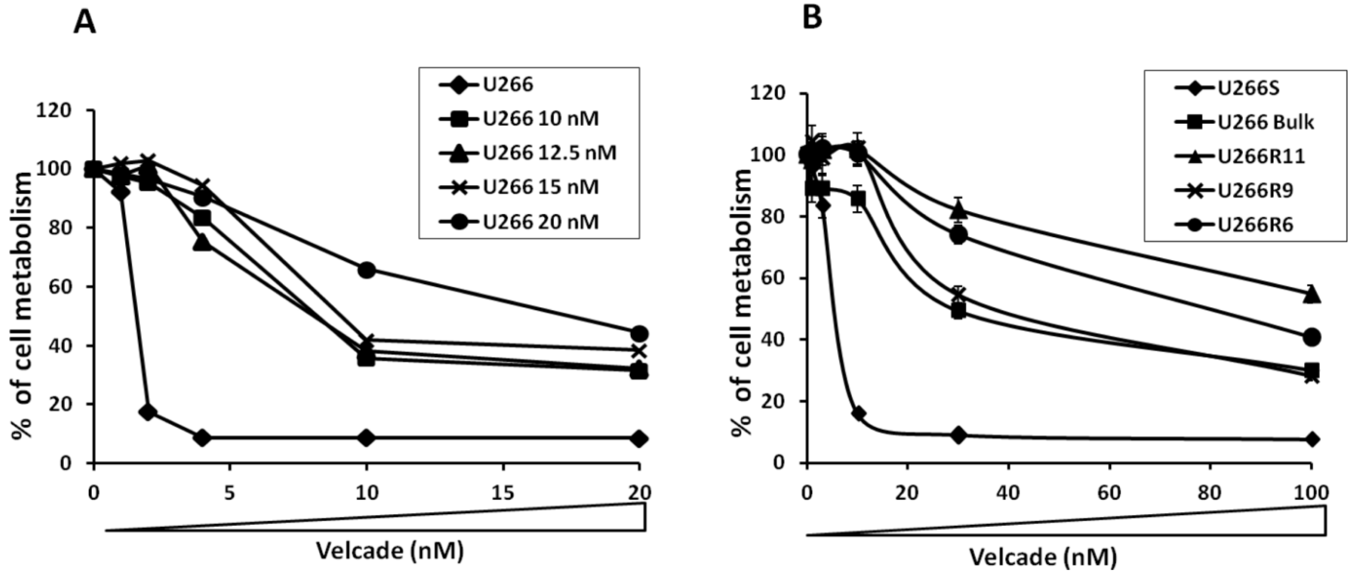

Supplementary figure 1: Establishment of a cell line resistant to velcade. From the parental U266 cell line, we established resistant clones by addition in the culture medium of increasing concentrations of velcade (up to 20 nM) for 10 months. **A,** The cells corresponding to the different steps of the selection with velcade (10, 12.5, 15 and 20 nM) were stimulated with increasing concentrations of velcade (1 to 20 nM). Cell viability was assessed with the XTT cell metabolism assay. **B,** From the bulk corresponding to the final step of incubation with velcade (20 nM), we selected 3 representative clones by limiting dilution (U266R6, U266R9 and U266R11). Parental U266 cells and the bulk, and the 3 representative clones were collected and incubated with increasing concentrations of velcade (1 to 100 nM). Cell viability was assessed with the XTT cell metabolism assay.

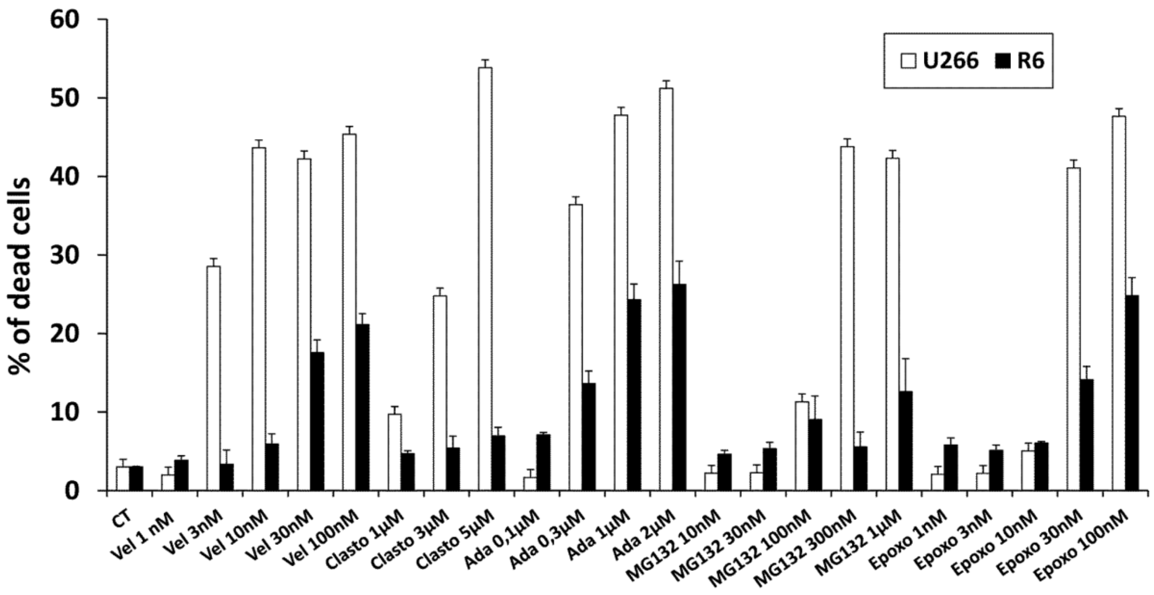

Supplementary figure 2: R6 cells are resistant to a wide panel of proteasome inhibitors. U266 and R6 cells were incubated for 48 h with different proteasome inhibitors at the indicated concentrations. Then, cells were collected, incubated with Propidium iodide and the percentage of dead cells was assessed by flow cytometry.

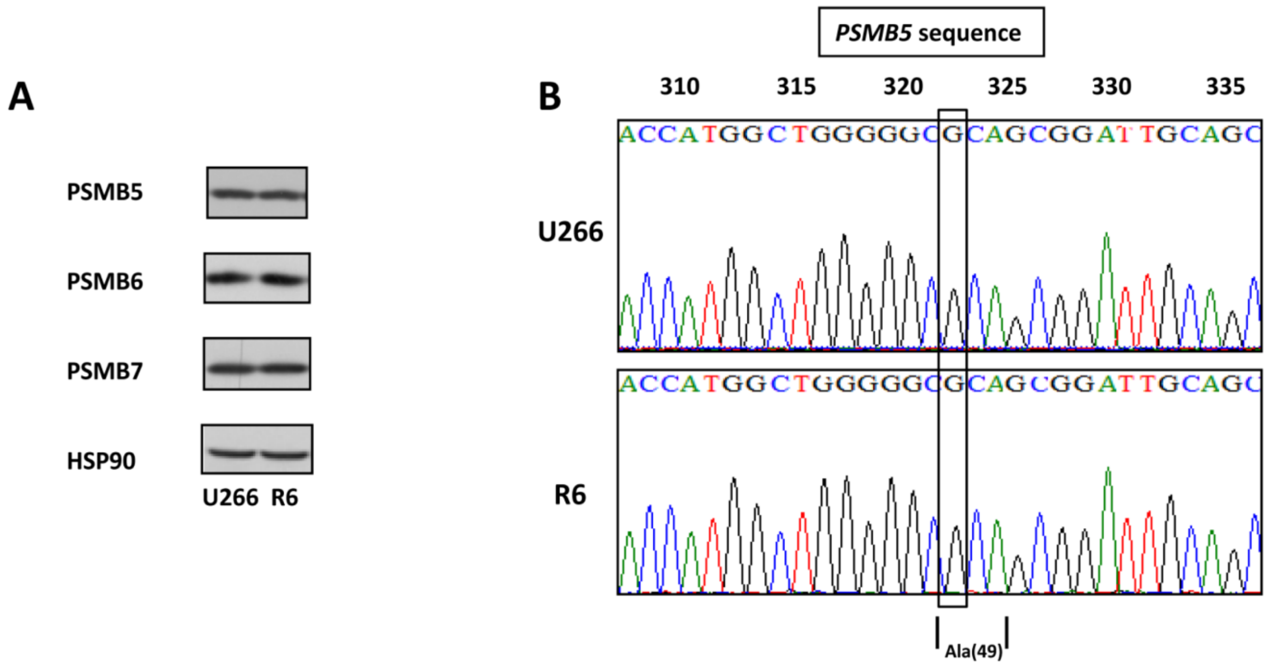

Supplementary figure 3: R6 cells have no apparent defect in proteasome subunits and activities. A, U266 and R6 cells were collected, washed and lysed, and protein extracts were subjected to SDS-PAGE and immunoblotting using anti-PSMB5, anti-PSMB6, anti-PSMB7 and anti-HSP90 antibodies. **B,** PSMB5 sequence was determined in the U266 and R6 cell lines. No mutation in guanine 322 was detected indicating the conservation of Ala49 in the R6 clone.

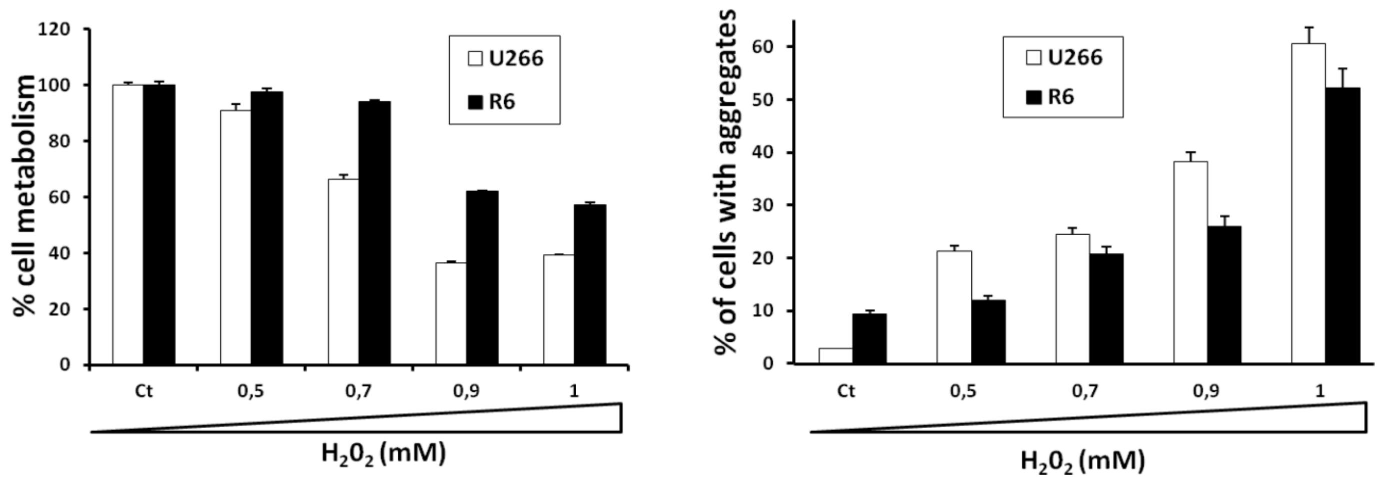

Supplementary figure 4: H_2O_2-treated R6 clone exhibits decreased accumulation of protein aggregates and partial resistance to cell death. U266 and R6 cells were incubated with increasing concentrations of H_2O_2 (0.5 to 1 mM) for 24 h. Then, the cells were collected and cell viability was assessed by an XTT cell metabolism assay (left panel). In parallel, the percentage of cells with aggregates was determined by flow cytometry (right panel).

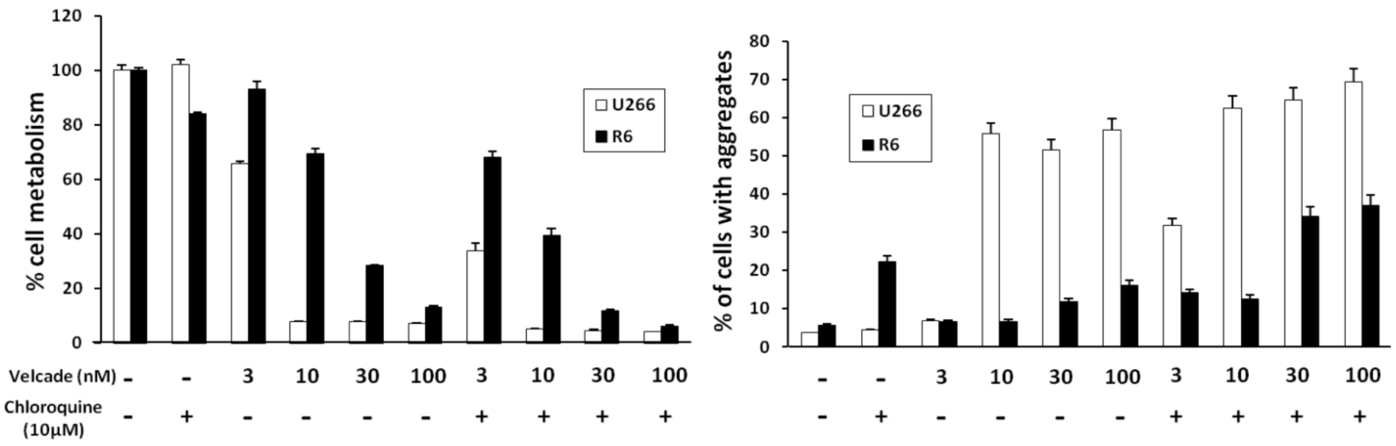

Supplementary figure 5: Inhibition of lysosomal degradation by chloroquine restores sensitivity to velcade in R6 cells. U266 and R6 cells were incubated with increasing concentrations of velcade (3 to 100 nM) in the presence or in absence of chloroquine (10 µM) for 24 h. Then, the cells were collected and cell viability was assessed by an XTT cell metabolism assay (left panel). In parallel, the percentage of cells with aggregates was determined by flow cytometry (right panel).

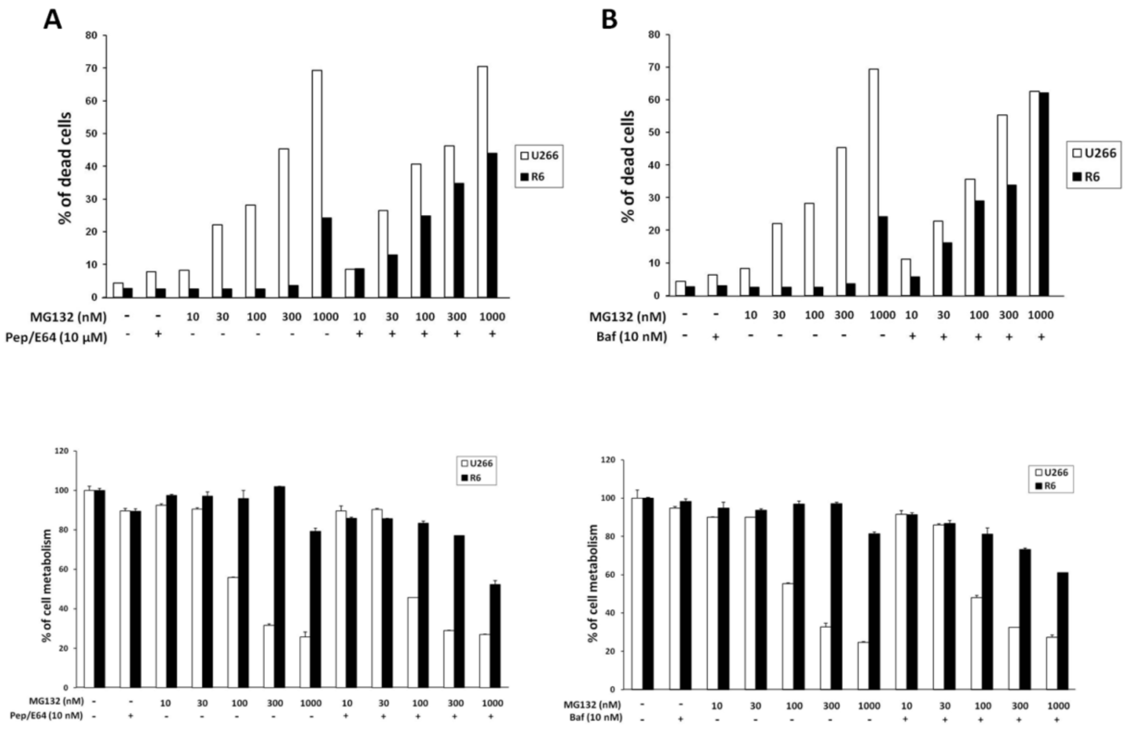

Supplementary figure 6: Inhibition of lysosomal degradation restores sensitivity to MG132 in R6 cells. U266 and R6 cells were incubated with increasing concentrations of MG132 (10 to 1000 nM) in the presence or in absence of the combination of either E64 and Pepstatin (10 µM) **(A)** or Bafilomycin A1 (10 nM) **(B)** for 24 h. Then, cells were collected, incubated with Propidium iodide and the percentage of dead cells was assessed by flow cytometry (upper panels). In parallel, cell viability was assessed by an XTT cell metabolism assay (lower panels).

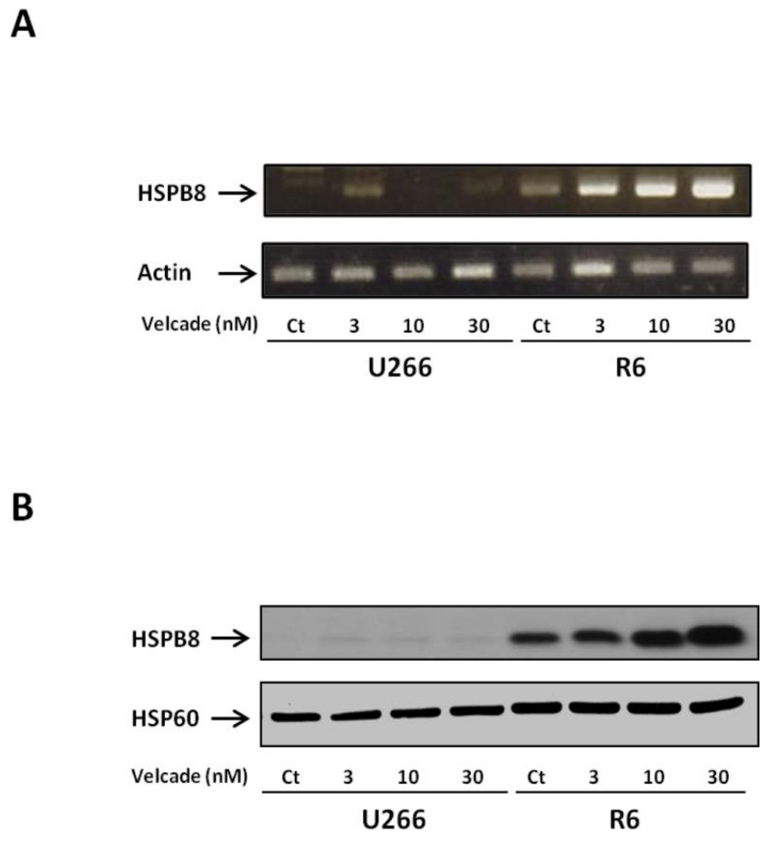

Supplemental figure 7: Velcade induces expression of HSPB8 at the mRNA and the protein level. U266 and R6 cells were incubated with increasing concentrations of velcade (3 to 30 nM) for 24 h. **A,** Then, cells were collected and total RNAs were purified. HSPB8 and Actin transcripts were amplified by RT-PCR and separated in agarose gel. **B,** In parallel, cells were collected, washed and lysed, and protein extracts were subjected to SDS-PAGE and immunoblotting using anti-HSPB8 and HSP60 antibodies.

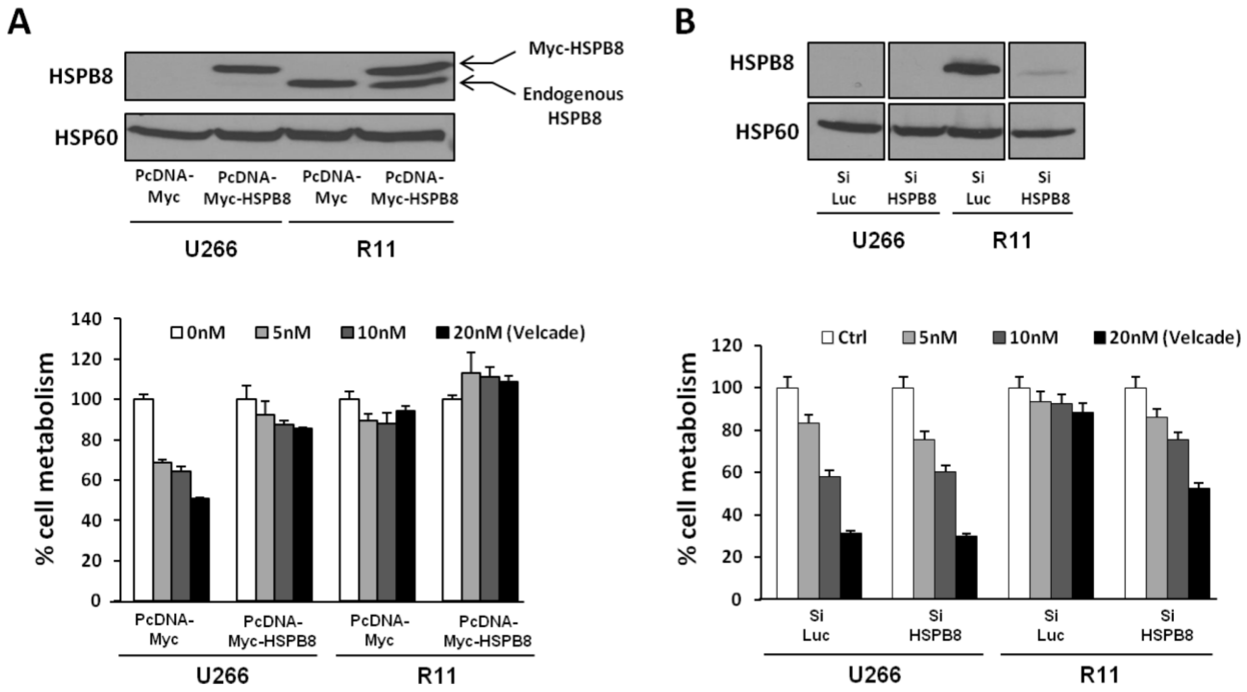

Supplemental figure 8: HSPB8 overexpression in R11 cells is responsible for the resistance to velcade.A, U266 and R11 cells were transfected with either control PcDNA3-Myc or PcDNA3-Myc-HSPB8 vectors. 48 h later, one fraction of the cells were harvested and lysed. Exogenous Myc-HSPB8 protein (upper band) and endogenous HSPB8 protein (lower band) level was quantified by western blotting using anti-HSPB8 antibody. Correct normalization of protein extracts was confirmed using anti-HSP60 antibody (upper panel). The second fraction of the cells was stimulated with increasing concentrations of velcade (5 to 20 nM) for 24 h. Then, the cells were collected and cell viability was assessed by the XTT cell metabolism assay (lower panel). **B,** U266 and R9 were transfected with either control siRNA or with HSPB8 siRNA. 48h later, one fraction of the cells was collected and lysed, and HSPB8 protein silencing

was confirmed by western blotting using anti-HSPB8 antibody. Normalization of protein extracts was confirmed using anti-HSP60 antibody (upper panel). The second portion of the cells was stimulated with increasing concentrations of velcade (5 to 20 nM) for 24 h. Cell viability was assessed using the XTT cell metabolism assay (lower panel).

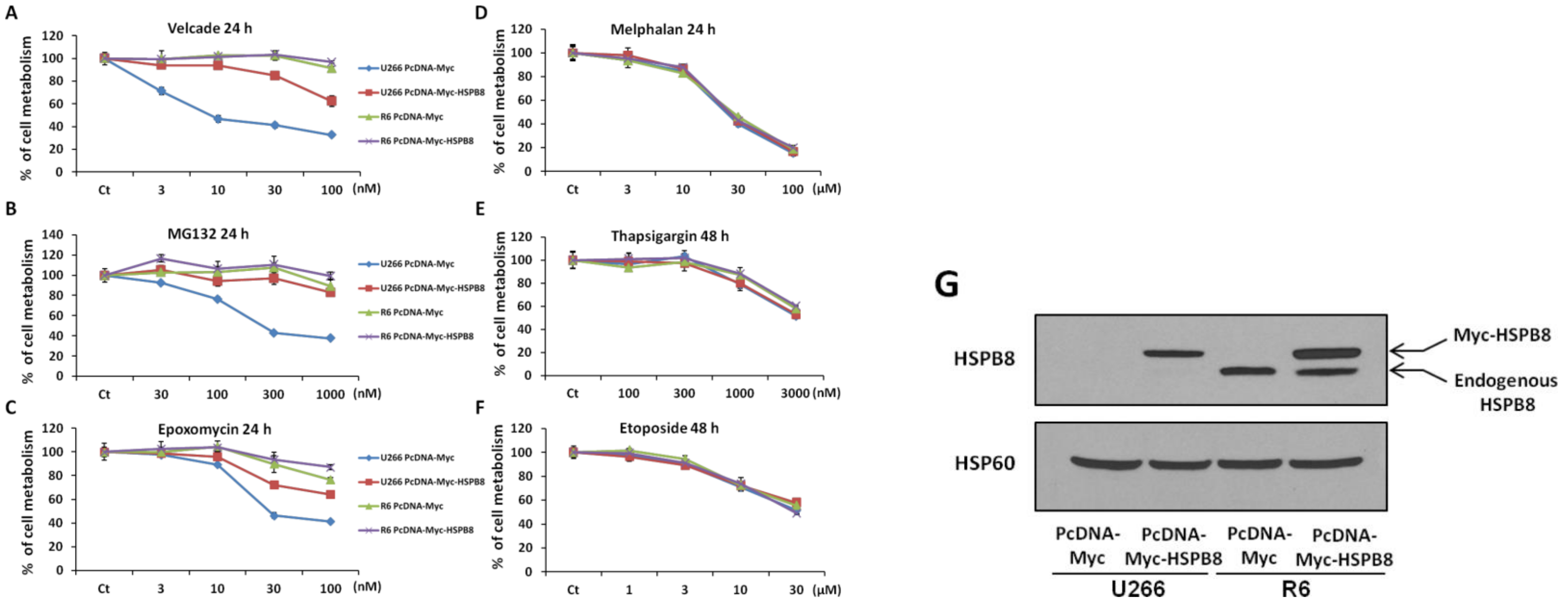

Supplemental figure 9: HSPB8 overexpression protects U266 cells to different proteasome inhibitors but not to various proapoptotic stimuli. A to F, U266 and R6 cells were transfected with either control PcDNA3-Myc or PcDNA3-Myc-HSPB8 vectors. 48 h later, cells were stimulated with increase concentrations of proteasome inhibitors (Velcade, MG132 and Epoxomycin) or various proapoptotic stimuli (Melphalan, Thapsigargin and Etoposide) at indicated time. Then, the cells were collected and cell viability was assessed by the XTT cell metabolism assay. **G,** In parallel, one fraction of the cells were

harvested and lysed. Exogenous Myc-HSPB8 protein (upper band) and endogenous HSPB8 protein (lower band) level was quantified by western blotting using anti-HSPB8 antibody. Correct normalization of protein extracts was confirmed using anti-HSP60 antibody.

Table S1: List of the best 160 RefSeq annotated transcripts significantly modulated in the R6 clone and the bulk population compared to U266 parental cells (absolute log2 Ratio above 2). Affymetrix probe set ID, NCBI RefSeq IDs give access to transcripts annotations. Logarithm (base 2) of the average intensity and logarithm (base 2) of the ratio are represented.

Probe Set ID	Gene Symbol	Accession	Gene Description	Log2 Average	Log2 (R6/U266)	Log2 (Bulk/U266)
8101675	ABCG2	NM_004827	ATP-binding cassette, sub-family G (WHITE), member 2	7.06	2.07	0.93
8115490	ADAM19	NM_033274	ADAM metallopeptidase domain 19 (meltrin beta)	6.91	1.97	2.79
8145317	ADAMDEC1	NM_014479	ADAM-like, decysin 1	7.04	-3.73	-1.30
8052882	ADD2	NM_017488	adducin 2 (beta)	7.76	-2.58	-2.44
7962183	AK3L1	NM_001005353	adenylate kinase 3-like 1	6.52	-1.19	2.39
7968344	ALOX5AP	NM_001629	arachidonate 5-lipoxygenase-activating protein	8.62	2.24	3.22
8075695	APOL3	NR_027833	apolipoprotein L, 3	6.33	-2.27	-1.66
8072710	APOL6	NM_030641	apolipoprotein L, 6	7.98	-2.52	-1.03
7961532	ARHGDIB	NM_001175	Rho GDP dissociation inhibitor (GDI) beta	6.66	2.19	2.09
7927599	ASAH2	NM_019893	N-acylsphingosine amidohydrolase (non-lysosomal ceramidase) 2	6.77	1.61	2.60
7926875	BAMBI	NM_012342	BMP and activin membrane-bound inhibitor homolog (Xenopus laevis)	7.16	-2.96	-2.76
8104601	BASP1	NM_006317	brain abundant, membrane attached signal protein 1	7.76	2.19	1.41
8174201	BEX1	NM_018476	brain expressed, X-linked 1	6.66	3.10	3.66
7963289	BIN2	NM_016293	bridging integrator 2	6.59	0.80	2.63
7978776	C14orf106	NM_018353	chromosome 14 open reading frame 106	5.97	-1.18	-2.12
8118324	C2	NM_000063	complement component 2	5.91	-2.48	-0.98
8118409	C4A	NM_007293	complement component 4A (Rodgers blood group)	7.67	-2.33	-0.27
8139921	CALN1	NM_001017440	calneuron 1	7.00	0.96	2.14

8107307	CAMK4	NM_001744	calcium/calmodulin-dependent protein kinase IV	7.66	2.11	2.25
8137874	CARD11	NM_032415	caspase recruitment domain family, member 11	6.80	2.26	2.14
7951397	CASP1	NM_033292	caspase 1, apoptosis-related cysteine peptidase (interleukin 1, beta, convertase)	5.57	-2.36	-1.66
7899160	CD52	NM_001803	CD52 molecule	9.88	1.37	2.24
7903893	CD53	NM_000560	CD53 molecule	9.27	-4.69	-2.56
8111255	CDH10	NM_006727	cadherin 10, type 2 (T2-cadherin)	5.14	-3.10	-3.13
8158976	CEL	NM_001807	carboxyl ester lipase (bile salt-stimulated lipase)	7.09	-2.02	-1.64
8165974	CLCN4	NM_001830	chloride channel 4	6.39	2.79	2.67
7961102	CLEC1B	NM_016509	C-type lectin domain family 1, member B	5.47	-3.11	-2.86
8077299	CNTN6	NM_014461	contactin 6	5.72	-3.41	-3.39
8100127	COX7B2	NM_130902	cytochrome c oxidase subunit VIIb2	5.92	2.60	1.95
7926127	CUGBP2	NM_001025077	CUG triplet repeat, RNA binding protein 2	6.88	-2.15	0.09
8166690	CXorf59	BC101698	chromosome X open reading frame 59	7.62	2.10	1.52
8166440	DDX53	NM_182699	DEAD (Asp-Glu-Ala-Asp) box polypeptide 53	6.73	-2.50	-1.64
7923131	DENND1B	NM_001142795	DENN/MADD domain containing 1B	6.36	3.03	1.40
8142345	DOCK4	NM_014705	dedicator of cytokinesis 4	6.31	1.61	2.09
8153959	DOCK8	NM_203447	dedicator of cytokinesis 8	6.70	3.50	3.25
8082075	DTX3L	NM_138287	deltex 3-like (Drosophila)	7.39	-4.00	-3.40
7972157	EDNRB	NM_001122659	endothelin receptor type B	8.51	-4.03	-6.16
7980636	EML5	NM_183387	echinoderm microtubule associated protein like 5	5.42	-2.59	-2.72
8129497	EPB41L2	NM_001431	erythrocyte membrane protein band 4.1-like 2	6.22	2.88	3.15
8168678	FAM133A	BC113385	family with sequence similarity 133, member A	7.93	1.42	2.81
8096682	FLJ20184	NM_017700	hypothetical protein FLJ20184	5.96	-2.01	-1.83
8080714	FLNB	NM_001457	filamin B, beta	8.55	3.45	4.38
8100097	GABRG1	NM_173536	gamma-aminobutyric acid (GABA) A receptor, gamma 1	5.64	2.67	3.10
8167508	GAGE12B	NM_001127345	G antigen 12B	6.92	3.17	2.81
8167560	GAGE12C	NM_001098408	G antigen 12C	6.18	4.24	3.93
8167482	GAGE4	NM_001474	G antigen 4	6.96	3.30	2.99
7980580	GALC	NM_000153	galactosylceramidase	6.56	1.13	2.18

8116835	GCNT2	NM_145649	glucosaminyl (N-acetyl) transferase 2, I-branching enzyme (I blood group)	6.27	2.41	2.37
7961386	GPRC5D	NM_018654	G protein-coupled receptor, family C, group 5, member D	7.12	-1.40	-2.27
8101757	GPRIN3	NM_198281	GPRIN family member 3	7.49	-2.16	-2.03
8046906	GULP1	NM_016315	GULP, engulfment adaptor PTB domain containing 1	5.02	-1.04	-2.62
8124537	HIST1H3J	NM_003535	histone cluster 1, H3j	7.33	2.09	2.56
8124413	HIST1H4D	NM_003539	histone cluster 1, H4d	7.02	-2.07	-2.39
8042942	HK2	NM_000189	hexokinase 2	8.39	0.86	2.05
8125530	HLA-DMB	NM_002118	major histocompatibility complex, class II, DM beta	7.62	1.71	2.04
8125556	HLA-DPA1	NM_033554	major histocompatibility complex, class II, DP alpha 1	9.79	1.30	2.83
8178220	HLA-DPB1	NM_002121	major histocompatibility complex, class II, DP beta 1	8.04	1.52	2.84
8118556	HLA-DQA1	NM_002122	major histocompatibility complex, class II, DQ alpha 1	7.70	2.03	3.31
8178205	HLA-DQA2	NM_020056	major histocompatibility complex, class II, DQ alpha 2	7.16	2.14	2.79
8125447	HLA-DQB1	NM_002123	major histocompatibility complex, class II, DQ beta 1	7.81	1.67	2.79
8178193	HLA-DRA	NM_019111	major histocompatibility complex, class II, DR alpha	9.86	2.03	3.85
8125436	HLA-DRB5	NM_002125	major histocompatibility complex, class II, DR beta 5	7.56	1.53	3.27
7948982	HRASLS2	NM_017878	HRAS-like suppressor 2	7.30	-3.21	-2.83
7959102	HSPB8	NM_014365	heat shock 22kDa protein 8	6.13	2.29	1.95
7976443	IFI27	NM_001130080	interferon, alpha-inducible protein 27	7.90	-2.08	-3.88
7902553	IFI44	NM_006417	interferon-induced protein 44	5.98	-4.44	-6.94
7902541	IFI44L	NM_006820	interferon-induced protein 44-like	7.10	-5.20	-7.92
7914127	IFI6	NM_002038	interferon, alpha-inducible protein 6	10.18	-2.70	-3.21
7929065	IFIT1	NM_001548	interferon-induced protein with tetratricopeptide repeats 1	7.80	-3.66	-4.79
7929047	IFIT2	NM_001547	interferon-induced protein with tetratricopeptide repeats 2	6.68	-3.78	-3.78
7929052	IFIT3	NM_001031683	interferon-induced protein with tetratricopeptide repeats 3	8.18	-2.46	-3.25
7929072	IFIT5	NM_012420	interferon-induced protein with tetratricopeptide repeats 5	7.54	-1.81	-2.46
8080562	IL17RB	NM_018725	interleukin 17 receptor B	5.56	1.67	2.08
7994292	IL21R	NM_181078	interleukin 21 receptor	7.84	2.34	3.06
8130408	IPCEF1	NM_001130700	interaction protein for cytohesin exchange factors 1	5.41	-2.42	-2.62
7924058	IRF6	NM_006147	interferon regulatory factor 6	6.50	-2.61	-0.38

7979529	KCNH5	NM_139318	potassium voltage-gated channel, subfamily H (eag-related), member 5	5.32	2.48	2.88
8040458	KCNS3	NM_002252	potassium voltage-gated channel, delayed-rectifier, subfamily S, member 3	6.42	-2.81	-2.25
8099721	KIAA0746	NM_015187	KIAA0746 protein	6.05	2.44	2.02
7963851	KIAA0748	NM_001098815	KIAA0748	5.27	2.18	1.60
7936529	KIAA1598	NM_001127211	KIAA1598	6.84	3.03	2.23
8039257	LAIR1	NM_002287	leukocyte-associated immunoglobulin-like receptor 1	6.11	0.91	2.04
8092348	LAMP3	NM_014398	lysosomal-associated membrane protein 3	7.61	-2.62	-2.74
7914270	LAPTM5	NM_006762	lysosomal protein transmembrane 5	8.67	3.52	5.10
7902353	LHX8	NM_001001933	LIM homeobox 8	6.02	2.19	2.53
8040483	LOC100129278	ENST00000405799	similar to tudor domain containing 1	6.49	-1.64	-2.48
8117243	LRRC16A	NM_017640	leucine rich repeat containing 16A	6.49	-2.19	-1.24
8089714	LSAMP	NM_002338	limbic system-associated membrane protein	7.83	-2.95	-1.56
8056113	LY75	NM_002349	lymphocyte antigen 75	5.77	2.34	3.90
7907032	MAEL	NM_032858	maelstrom homolog (Drosophila)	6.98	6.65	6.29
8166669	MAGEB16	NM_001099921	melanoma antigen family B, 16	7.22	3.72	3.82
8175562	MAGEC2	NM_016249	melanoma antigen family C, 2	6.71	-3.31	-0.12
7983228	MAP1A	NM_002373	microtubule-associated protein 1A	8.27	3.40	2.92
8047926	MAP2	NM_002374	microtubule-associated protein 2	5.93	-4.42	-3.15
8113551	MCC	NM_001085377	mutated in colorectal cancers	6.35	-1.70	-2.07
8129573	MOXD1	NM_015529	monooxygenase, DBH-like 1	5.44	2.10	1.45
8131927	MPP6	NM_016447	membrane protein, palmitoylated 6 (MAGUK p55 subfamily member 6)	7.48	2.03	0.89
7947156	MUC15	NM_001135091	mucin 15, cell surface associated	5.06	-3.47	-1.23
8068713	MX1	NM_002462	myxovirus (influenza virus) resistance 1, interferon-inducible protein p78 (mouse)	9.50	-3.55	-3.88
8173917	NAP1L3	NM_004538	nucleosome assembly protein 1-like 3	5.90	2.83	2.59
7943892	NCAM1	NM_181351	neural cell adhesion molecule 1	5.88	-2.36	-2.89
7958913	OAS2	NM_002535	2'-5'-oligoadenylate synthetase 2, 69/71kDa	9.85	-2.05	-2.20
7958895	OAS3	NM_006187	2'-5'-oligoadenylate synthetase 3, 100kDa	8.85	-2.36	-2.29

8105908	OCLN	NM_002538	occludin	5.04	-2.83	-3.06
8117622	OR2B6	NM_012367	olfactory receptor, family 2, subfamily B, member 6	5.21	-0.87	-2.98
8082100	PARP14	NM_017554	poly (ADP-ribose) polymerase family, member 14	8.01	-3.83	-3.24
8090018	PARP9	NM_031458	poly (ADP-ribose) polymerase family, member 9	7.03	-4.39	-4.27
8086961	PFKFB4	NM_004567	6-phosphofructo-2-kinase/fructose-2,6-biphosphatase 4	7.44	-0.31	2.08
8135363	PIK3CG	NM_002649	phosphoinositide-3-kinase, catalytic, gamma polypeptide	7.08	4.45	4.45
8147012	PKIA	NM_006823	protein kinase (cAMP-dependent, catalytic) inhibitor alpha	5.91	2.12	0.89
8042391	PLEK	NM_002664	pleckstrin	5.62	0.66	2.87
8142997	PLXNA4	NM_020911	plexin A4	7.17	-3.97	-4.11
8141076	PON2	NM_000305	paraoxonase 2	7.74	1.33	2.01
8114900	PPP2R2B	NM_004576	protein phosphatase 2 (formerly 2A), regulatory subunit B, beta isoform	7.41	-4.16	-4.71
8135378	PRKAR2B	NM_002736	protein kinase, cAMP-dependent, regulatory, type II, beta	6.62	3.03	2.11
7927606	PRKG1	NM_001098512	protein kinase, cGMP-dependent, type I	5.59	-2.05	-1.07
8163795	PSMD5	NM_005047	proteasome (prosome, macropain) 26S subunit, non-ATPase, 5	5.83	-4.29	-4.13
8148501	PTP4A3	NM_032611	protein tyrosine phosphatase type IVA, member 3	8.08	-2.10	-1.57
8046428	RAPGEF4	NM_007023	Rap guanine nucleotide exchange factor (GEF) 4	7.29	-2.76	-2.58
8064790	RASSF2	NM_014737	Ras association (RalGDS/AF-6) domain family member 2	5.81	2.35	1.87
7922717	RGS16	NM_002928	regulator of G-protein signaling 16	8.15	2.22	2.14
8061247	RIN2	NM_018993	Ras and Rab interactor 2	6.79	-2.38	-2.09
7922707	RNASEL	NM_021133	ribonuclease L (2',5'-oligoisoadenylate synthetase-dependent)	6.67	2.28	1.64
8040080	RSAD2	NM_080657	radical S-adenosyl methionine domain containing 2	6.48	-2.41	-3.81
7920123	S100A10	NM_002966	S100 calcium binding protein A10	7.95	-2.32	0.36
7922550	SEC16B	NM_033127	SEC16 homolog B (S. cerevisiae)	6.89	-2.75	-2.59
8140668	SEMA3A	NM_006080	sema domain, immunoglobulin domain (Ig), short basic domain, secreted, (semaphorin) 3A	6.42	-3.11	-2.60
8059376	SERPINE2	NM_001136529	serpin peptidase inhibitor, clade E (nexin, plasminogen activator inhibitor type 1), member 2	5.54	1.91	2.24
7956658	SLC16A7	NM_004731	solute carrier family 16, member 7 (monocarboxylic acid transporter 2)	5.89	-3.57	-3.28
8091260	SLC9A9	NM_173653	solute carrier family 9 (sodium/hydrogen exchanger), member	5.94	2.08	2.56

			9			
7986214	SLCO3A1	NM_013272	solute carrier organic anion transporter family, member 3A1	8.48	-2.71	-2.33
8171883	SMEK3P	NR_002784	SMEK homolog 3, suppressor of mek1 (Dictyostelium) pseudogene	6.04	3.25	3.70
7981992	SNORD116-22	NR_003336	small nucleolar RNA, C/D box 116-22	5.29	2.26	0.57
8115327	SPARC	NM_003118	secreted protein, acidic, cysteine-rich (osteonectin)	6.52	3.49	3.41
8096301	SPP1	NM_001040058	secreted phosphoprotein 1	7.19	5.16	4.85
8172787	SSX2	NM_003147	synovial sarcoma, X breakpoint 2	6.36	3.38	3.14
8167261	SSX4	NM_005636	synovial sarcoma, X breakpoint 4	6.21	1.95	2.55
8066822	SULF2	NM_018837	sulfatase 2	7.52	1.80	2.42
7986195	SV2B	NM_014848	synaptic vesicle glycoprotein 2B	5.43	2.28	3.01
8053753	TEKT4	AK097438	tektin 4	7.56	-1.57	-2.13
8081799	TIGIT	NM_173799	T cell immunoreceptor with Ig and ITIM domains	5.80	2.04	1.48
8170834	TKTL1	NM_012253	transketolase-like 1	7.30	2.44	2.56
8161701	TMEM2	NM_013390	transmembrane protein 2	7.42	-1.84	-2.90
8008547	TOM1L1	NM_005486	target of myb1 (chicken)-like 1	7.37	-2.83	-2.48
8071155	USP18	NM_017414	ubiquitin specific peptidase 18	7.46	-3.17	-3.89
8043197	VAMP8	NM_003761	vesicle-associated membrane protein 8 (endobrevin)	7.66	-2.26	-1.65
8176650	VCY	NM_004679	variable charge, Y-linked	7.45	1.71	2.56
7910971	WDR64	NM_144625	WD repeat domain 64	5.64	-2.05	-2.62
8088180	WNT5A	NM_003392	wingless-type MMTV integration site family, member 5A	5.10	1.23	3.23
8004184	XAF1	NM_017523	XIAP associated factor 1	7.15	-3.06	-4.07
8089701	ZBTB20	NM_015642	zinc finger and BTB domain containing 20	5.69	1.56	2.60
8055624	ZEB2	NM_014795	zinc finger E-box binding homeobox 2	6.76	2.75	3.22
8027368	ZNF254	NM_203282	zinc finger protein 254	5.37	2.38	1.49
8115196	ZNF300	NM_052860	zinc finger protein 300	6.09	3.85	3.50
8033780	ZNF426	NM_024106	zinc finger protein 426	7.23	1.56	2.60
8027345	ZNF492	NM_020855	zinc finger protein 492	5.63	2.49	1.92
8035847	ZNF675	NM_138330	zinc finger protein 675	5.80	2.53	2.58
8035825	ZNF676	NM_001001411	zinc finger protein 676	5.93	3.68	5.45
8035855	ZNF681	NM_138286	zinc finger protein 681	5.50	2.43	3.13

8027348	ZNF730	AK131472	zinc finger protein 730	5.44	2.76	3.27
